# Travel-Related Antimicrobial Resistance: A Systematic Review

**DOI:** 10.3390/tropicalmed6010011

**Published:** 2021-01-16

**Authors:** Hamid Bokhary, Krisna N. A. Pangesti, Harunor Rashid, Moataz Abd El Ghany, Grant A. Hill-Cawthorne

**Affiliations:** 1 School of Public Health, The University of Sydney, Sydney, NSW 2006, Australia; kpan4827@uni.sydney.edu.au (K.N.A.P.); grant.hill-cawthorne@sydney.edu.au (G.A.H.-C.); 2 University Medical Center, Umm Al-Qura University, Al Jamiah, Makkah, Makkah Region 24243, Saudi Arabia; 3 The Marie Bashir Institute for Infectious Diseases and Biosecurity, The University of Sydney, Westmead, NSW 2145, Australia; harunor.rashid@health.nsw.gov.au (H.R.); or moataz.mohamed19@outlook.com (M.A.E.G.); 4 The Westmead Institute for Medical Research, Westmead, NSW 2145, Australia; 5 National Centre for Immunisation Research and Surveillance (NCIRS), Kids Research, The Children’s Hospital at Westmead, Westmead, NSW 2145, Australia; 6 The Westmead Clinical School, Faculty of Medicine and Health, The University of Sydney, Sydney, NSW 2006, Australia

**Keywords:** travel, antimicrobial resistance, medical traveller, enteric bacteria, multidrug resistance

## Abstract

There is increasing evidence that human movement facilitates the global spread of resistant bacteria and antimicrobial resistance (AMR) genes. We systematically reviewed the literature on the impact of travel on the dissemination of AMR. We searched the databases Medline, EMBASE and SCOPUS from database inception until the end of June 2019. Of the 3052 titles identified, 2253 articles passed the initial screening, of which 238 met the inclusion criteria. The studies covered 30,060 drug-resistant isolates from 26 identified bacterial species. Most were enteric, accounting for 65% of the identified species and 92% of all documented isolates. High-income countries were more likely to be recipient nations for AMR originating from middle- and low-income countries. The most common origin of travellers with resistant bacteria was Asia, covering 36% of the total isolates. Beta-lactams and quinolones were the most documented drug-resistant organisms, accounting for 35% and 31% of the overall drug resistance, respectively. Medical tourism was twice as likely to be associated with multidrug-resistant organisms than general travel. International travel is a vehicle for the transmission of antimicrobial resistance globally. Health systems should identify recent travellers to ensure that adequate precautions are taken.

## 1. Introduction

Antimicrobial resistance (AMR) is a growing public health burden that is a serious threat to global health security [1]. The World Health Organization (WHO) has emphasised the broad impact that AMR will have on human lives, including on health, economic prosperity and other livelihoods [2]. A number of reports have now highlighted the substantially increased levels of AMR bacteria present in many regions of the world [2,3]. For example, the US Centers for Disease Control and Prevention (CDC) have estimated that there are around 2 million infectious cases in the US annually that are resistant to at least one antimicrobial, resulting in about 23,000 deaths and costing the US health system US$20–$35 billion [4]. Importantly, it has been estimated that this increase in the emergence of AMR organisms can also increase the morbidity and mortality of infectious diseases, as it hampers the ability of antimicrobial drugs to cure infections [3,5]. AMR infections currently result in 700,000 global deaths every year, with associated mortality estimated to claim 10 million lives per year by 2050 [5]. These high mortality rates associated with AMR are expected to cause a cumulative loss of around US$100 trillion to the total world gross domestic product (GDP) in 2050 [5].

The challenges of AMR are complex and multifaceted, with multiple drivers present and interlinked between different hosts and ecologies, including humans, animals, food and the environment [6,7]. These multiple links allow for the movement of by-products of antimicrobial drugs, AMR bacteria, and mobile genetic elements or AMR genes (ARGs), among and between these ecologies, all of which enhance the dissemination of AMR [8]. At the same time, the continuous movement of people across the globe also plays a key role in the emergence and dissemination of AMR organisms [9]. Recently, many studies highlighted the impact of overuse and misuse of antibiotics [10,11], the paucity of antibiotic development [12], and poor access to quality and affordable antibiotics and diagnostics [13,14] in promoting the global transmission of AMR bacteria.

In the past few decades, there has been significant increase in the number of international travellers, mostly of tourists [15]. The United Nations World Tourism Organization (UNWTO) has reported that 19% of the world’s population (1.4 billion people) travelled across international borders in 2018 [16]. It is estimated that 11 million individuals have travelled for medical tourism to seek affordable healthcare overseas [17]. In addition, during the past two decades, there has been a very substantial increase in the number of forcibly displaced people globally, particularly those who have been forced to travel from conflict regions in Africa and Asia [18]. Recently, there has been growing evidence that this migration of forcibly displaced people can contribute to the global transmission of AMR [19,20,21].

Historically, globalisation and human migration have profoundly contributed to the emergence and dissemination of infectious diseases [22]. For example, cholera and meningococcal meningitis outbreaks have been associated with international travellers returning from affected (endemic) areas [23,24]. Various international regulations and health protocols have been developed to reduce the burden of such diseases by focusing on vaccination and/or chemoprophylaxis [25,26]; consequently, the risk of travellers returning with colonisation of or infection with AMR organisms has also increased during the past two decades, with the magnitude of this risk varying according to the origin and destinations of travel [9]. The carriage of AMR bacteria following travel to highly endemic AMR regions has been shown to persist for up to 12 months post-travel, which amplifies the risk of introducing AMR organisms into susceptible populations [27].

Here, we systematically review the literature to identify the impact of planned and desired international travel on the global dissemination of AMR in order to better understand the key risk factors that promote the transmission of AMR and to assist health authorities in planning and predicting how to strengthen their health systems in response to the movement of drug-resistant infections.

## 2. Materials and Methods

### 2.1. Search Strategy

**Figure 1:**
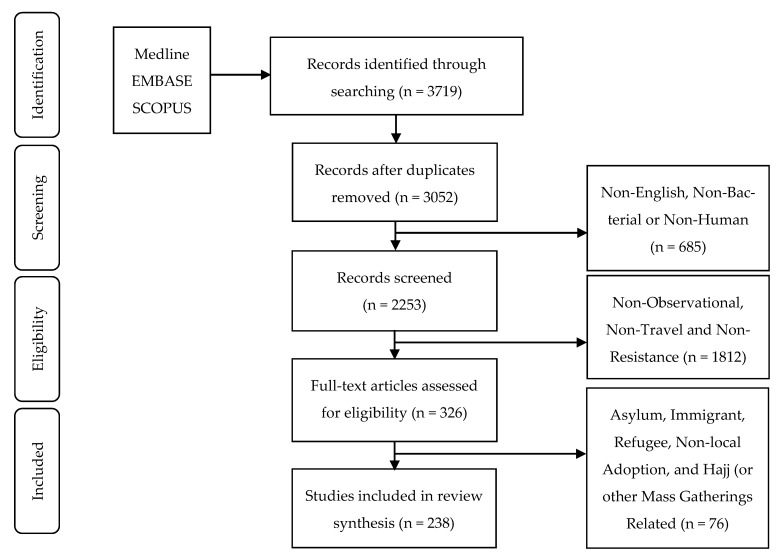
Preferred reporting items for systematic reviews and meta-analyses (PRISMA) flow diagram for the current systematic review: the methods used for search, identification, screening and the selection process for our review.

Studies and reports were identified by searching electronic databases, including Medline (PubMed and Ovid), Embase and Scopus, from database inception to the end of June 2019. The search results are presented as per the preferred reporting items for systematic reviews and meta-analyses (PRISMA) guidelines [28] in Figure 1. We used a combination of key words including: “travel” OR “pilgrim*” OR “Hajj” (also alternative spellings “Hadj” or “Haj”) OR “Olympic” OR “overseas student” OR “international student” OR “immigrant” OR “world cup” OR “mass gathering” OR “crowding” OR “tourism” OR “travel medicine” OR “holiday” AND “drug resistance” OR “methicillin-resistant *Staphylococcus aureus*” OR “antimicrobial resistan*”. In addition, a manual search was performed on the reference lists of included studies to identify additional potentially relevant papers.

### 2.2. Selection Criteria

Studies that were non-English (language), nonbacterial (organism) or nonhuman (host) were excluded. After screening, studies that did not address travel and AMR were excluded. In addition, studies that had the same or partially the same population with duplicated isolates were included [29,30,31,32] (but have had their isolates numbers adjusted) or completely removed [33,34] (if there was true duplication to the isolate profiles). Isolates were included if there was no duplication of information [35,36]. Reviews, editorials, comments and other non-observational articles were also excluded.

Initially, the intent was to include all contexts of human movement. While developing the manuscript, other systematic reviews were published for some human movement contexts, such as refugees and Hajj [19,37]; therefore, studies with these special contexts of human movement were excluded: asylum seekers, immigrants, refugees, non-local adoption and mass-gathering attendees.

### 2.3. Study Assessment

Non-randomised studies were assessed based on the Newcastle-Ottawa Scale (NOS) [38] for cohort and case-control studies. Cross-sectional studies and surveys were assessed with an adapted version of NOS that was used previously by Modesti and colleagues [39]. Moreover, case reports and case series were assessed using a NOS-adapted method that was described by Murad and colleagues [40]. Assessment was based on the respective scoring system, thus setting the maximum score to 9 for cohort and case-control assessments, 10 for cross-sectional studies and surveys, and 5–8 (depending on the study) for case reports and case series article assessment. A grade of “A” was given to randomised control trials (RCTs) of adequate sample size, and it was graded as “B” if the sample size was not adequate. Regarding observational and non-randomised studies, grade “A” was given for 75% or above (in reference to the maximum score), “B” was given for 50–74% and “C” was given for less than a 50% score on a NOS-based or adapted assessment.

### 2.4. Data Analysis and Visualisations

The total number of studies identified from the nominated databases was 3719 articles, of which 667 were duplicates. By skimming the titles, 799 articles were excluded for not being in English or because they focused on other non-bacterial organisms or on zoonotic hosts. The remaining 2253 articles were screened via their abstracts. Excluding non-observational studies and studies not addressing travel and AMR resulted in 326 eligible studies. The decision to exclude the aforementioned special types of human movement yielded the final 238 studies that were included in this analysis. All phenotypic and molecular confirmations for the acquisition of AMR during travel documented in these studies were included in the analysis. Figures were created using template mapchart.net (https://mapchart.net/), which are licensed under the “Creative Commons Attribution-ShareAlike 4.0 International License” (https://creativecommons.org/licenses/by-sa/4.0/).

## 3. Results

### 3.1. AMR Associated with Planned Travel

A total of 30,060 AMR bacterial isolates associated with 17,470 instances of planned international travel (at least one AMR organism documented per instance) was documented in the 238 studies [27,29,30,31,32,33,35,36,41,42,43,44,45,46,47,48,49,50,51,52,53,54,55,56,57,58,59,60,61,62,63,64,65,66,67,68,69,70,71,72,73,74,75,76,77,78,79,80,81,82,83,84,85,86,87,88,89,90,91,92,93,94,95,96,97,98,99,100,101,102,103,104,105,106,107,108,109,110,111,112,113,114,115,116,117,118,119,120,121,122,123,124,125,126,127,128,129,130,131,132,133,134,135,136,137,138,139,140,141,142,143,144,145,146,147,148,149,150,151,152,153,154,155,156,157,158,159,160,161,162,163,164,165,166,167,168,169,170,171,172,173,174,175,176,177,178,179,180,181,182,183,184,185,186,187,188,189,190,191,192,193,194,195,196,197,198,199,200,201,202,203,204,205,206,207,208,209,210,211,212,213,214,215,216,217,218,219,220,221,222,223,224,225,226,227,228,229,230,231,232,233,234,235,236,237,238,239,240,241,242,243,244,245,246,247,248,249,250,251,252,253,254,255,256,257,258,259,260,261,262,263,264,265,266,267,268,269,270] included in this analysis (Table 1). Most studies reported the detection of at least one AMR bacterial species after travel, while travelling or prior to the commencement of travel. There were 2 RCTs, 2 cohort, 13 case-control, 155 cross-sectional (or survey) and 66 case report/series study types. Regarding study assessment, there were 29 studies scored as grade A, 92 scored as B and 177 scored as C based on NOS or assessments adapted from it (Table 1).

The total pooled population of travellers screened was 632,704, of which 26.33% (n = 166,615) were female and the gender was not documented in 38.22% (241,829). The average representative age of case patients from the pooled studies, when available, was 38.9 years (range 0–103.2 years). A subpopulation of travellers experienced exposure to healthcare systems whilst travelling: hospitalised in, admitted to, repatriated from or seeking treatment (such as medical tourists) from healthcare facilities. Of these 11,089 “medical travellers”, 23.09% (n = 2560) were female and the gender was not documented in 52.51% (n = 5823). The average representative age for medical travellers was 51.4 years (range 0–99 years).

The analysis demonstrated that AMR organisms originated from 139 countries and travelled to 34 countries in total. In general, low- and middle-income countries (124 out of 139) were the source of most AMR organisms, while high-income countries (20 of 34) constituted the major recipients of resistant bacteria.

The countries and regions given in the articles included in the analysis were categorised into 11 groups according to their geographical locations (Table S2). The top regions from which the majority of AMR organisms were sourced or to which people travelled are provided in Tables S3 and S4, respectively. Briefly, 37.12% (n = 11,157; documented in 174 studies), 12.50% (n = 3757; 104 studies) and 10.66% (n = 3205; 59 studies) of the AMR organisms reported in the studied articles originated from Asia, Africa, and Central and South America, respectively (sources). The top source countries of AMR bacteria were India (n = 3602; 70 studies), Kenya (n = 1176; 12 studies), Thailand (n = 1012; 34 studies), Mexico (n = 630; 21 studies), Spain (n = 386; 21 studies), Jamaica (n = 363; 4 studies) and Egypt (n = 292; 28 studies). The source countries of 28.34% (n = 8520; 63 studies) of the AMR organisms reported were either missing or associated with travellers from multiple regions; 50.66% (n = 15,229; 138 studies), 30.81% (n = 9261; 57 studies) and 5.49% (n = 1650; 11 studies) of the AMR organisms reported were in travellers to countries located in Europe, North America and Oceania, respectively. The destination of 9.85% (n = 2962; 4 studies) of the resistant organisms reported were not provided. The largest recipient countries of AMR bacteria included the USA (n = 5449; 40 studies), Canada (n = 3812; 17 studies), the UK (n = 3656; 22 studies), Finland (n = 3382; 10 studies), Spain (n = 1993; 17 studies) and Australia (n = 1650; 11 studies).

### 3.2. AMR Bacteria Associated with Planned International Travel

The identified resistant organisms comprised 26 bacterial species that mostly included Gram-negative bacteria (e.g., *Escherichia coli* and *Salmonella* spp.) and other bacterial species that are commonly associated with hospital- (e.g., *Staphylococcus aureus* and *Acinetobacter baumanii*) or community-acquired infections (Table S5). The bacterial species were not specified in 17.49% (n = 5258; 29 studies) of the resistant profiles reported, with enteric bacteria constituting 98.90% (n = 5200; 29 studies) of this group as a generalization. *Salmonella* spp. (20.07%, n = 6032; 63 studies), *Shigella* spp. (23.06%, n = 6931; 29 studies), *E. coli* (18.17%, n = 5461; 59 studies), *Campylobacter* spp. (10.91%, n = 3281; 19 studies) and S. *aureus* (7.19%, n = 2162; 35 studies) were the most common AMR bacteria reported.

Many of the drug groups for which resistance was reported are clinically important. Resistance to quinolones was documented in 9213 isolates, that to sulphonamides and trimethoprim was documented in 7268 isolates and that to cephalosporins was documented in 2100 isolates (Table S6). Resistance to beta-lactams was seen in 10,474 isolates, which includes resistance to penicillins in 6320 isolates and to carbapenems in 1922 isolates. This means that many of the AMR bacteria detected include species on the WHO list priority pathogen list of bacteria that pose the greatest threat to human health and resistance to critically important antibiotics [2]. These include carbapenem-resistant and extended-spectrum beta-lactamase (ESBL) producers of various *Enterobacteriaceae* members (e.g., *E. coli* and *Klebsiella* spp.), methicillin-resistant *S. aureus* (MRSA), and fluoroquinolone-resistant *Salmonella* spp. and *Campylobacter* spp. Some of the organisms on the WHO list could be detected over a number of decades, with Table 2 listing the detection rates of these nominated organisms by time period and location.

Some bacterial species have been the focus of previous studies on travel-related infections. However, some of these species were only occasionally detected in this study, including *Mycobacterium* spp. (n = 91; 5 studies), *Neisseria gonorrhoeae* (n = 120; 3 studies), *Pseudomonas* spp. (n = 44; 12 studies), *Burkholderia pseudomallei* (n = 5; 5 studies), or those that can cause epidemics (e.g., *Vibrio cholerae* (n = 5; 3 studies)) (Table S5). Nearly all AMR *M. tuberculosis* (n = 88; 4 studies) that were associated with travel originated from Africa and are resistant to both isoniazid and rifampicin (n = 86; 2 studies). Travel-associated AMR *B. pseudomallei* infections were sporadic, with the cases reported usually associated with travel to tropical areas. All travel-related AMR *N. gonorrheae* isolates were resistant to ciprofloxacin (n = 119; 2 studies).

### 3.3. Trends in the Movements of Travel-Associated AMR Bacteria

**Figure 2:**
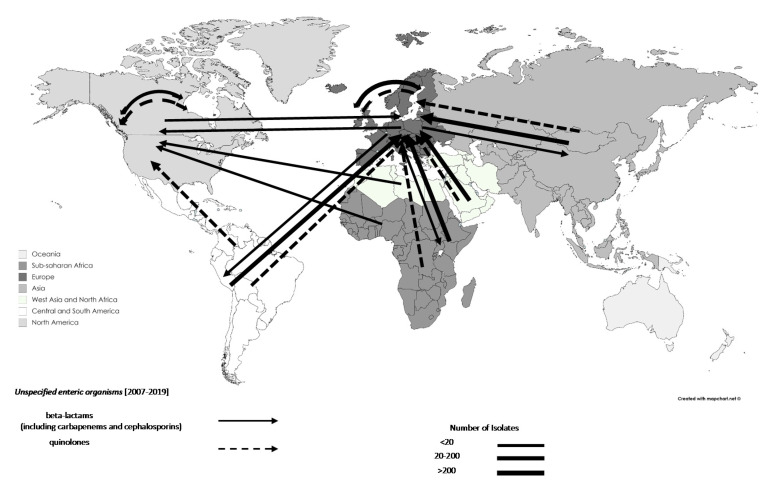
Travel-related antimicrobial resistance for unspecified enteric organisms’ movement, 2007–2019: data are shown by arrows representing antimicrobial resistant isolate movements, where the arrowhead represents the destination and the base of the arrow represents the source. Thus, double-headed arrows represent movements between the same regions. Different regions are represented by different shades.

Of the recorded AMR movements associated with travel, 91.83% (n = 27,593; 195 studies) were of enteric bacteria, of which the species was not identified in 18.85% (n = 5200). Of these unidentified enteric bacteria, 2448 were recorded between 1990 and 1999 (47.08%), 415 were recorded between 2000 and 2009 (7.98%), 2337 were recorded between 2010 and 2019 (44.75%), and none were recorded before 1990; the movements of these unidentified enteric bacteria are illustrated in Figure 2. A gradual increase was seen in the number of AMR enteric bacteria overall over time, from 1074 before 1990 (3.89%) to 6427 (1990–1999; 23.29%) and 15,067 AMR enteric bacteria (2000–2009; 54.60%). The majority of these AMR enteric bacteria were associated with travel originating from Asia (35.24%, n = 9725; excluding West Asia), and Central and South America (13.13%, n = 3623). Interestingly, 14.91% (n = 4113; 102 studies) of enteric bacteria associated with travel were categorised as multidrug-resistant (MDR, resistance to three or more antimicrobial classes). The AMR profiles for 37.66% (n = 1549) of MDR enteric bacteria associated with travel were available; 68.11% of MDR enteric bacteria (n = 1055) were resistant to beta-lactams (including carbapenems and cephalosporins) and sulphonamides (including trimethoprim), of which 32.99% (n = 348), 25.78% (n = 272) and 29.38% (n = 310) were co-resistant with amphenicols, quinolones or both, respectively.

**Figure 3:**
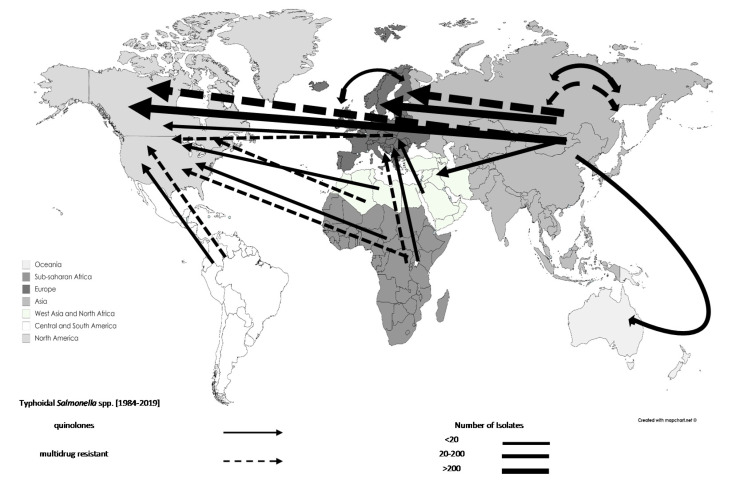
Travel-related antimicrobial resistant typhoidal *Salmonella* spp. movements, 1984–2019: data are shown by arrows representing antimicrobial resistant isolate movements, where the arrowhead represents the destination and the base of the arrow represents the source. Thus, double-headed arrows represent movements between the same regions. Different regions are represented by different shades.

**Figure 4:**
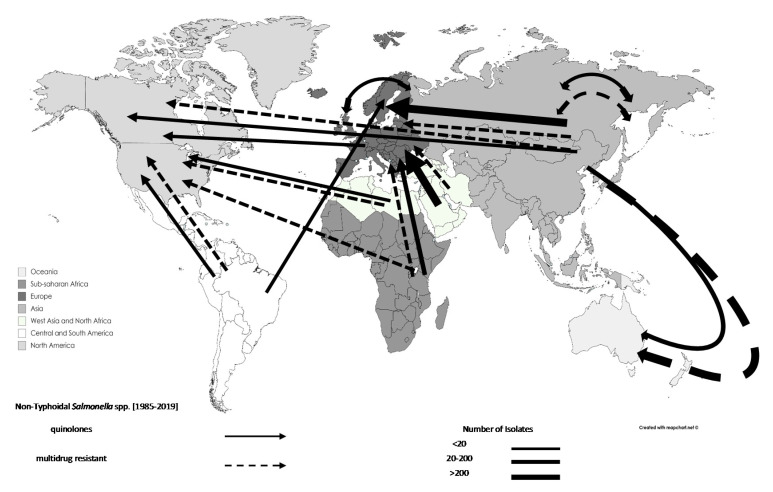
Travel-related antimicrobial resistant nontyphoidal *Salmonella* spp. movements, 1990–2019: data are shown by arrows representing antimicrobial resistant isolate movements, where the arrowhead represents the destination and the base of the arrow represents the source. Thus, double-headed arrows represent movements between the same regions. Different regions are represented by different shades.

The analysis demonstrated an increase in the total number of resistant *Salmonella* spp. associated with travel from 1553 in 1990–1999 (25.75%) to 3549 in 2000–2009 (58.84%). Specifically, the rates of reporting quinolone-resistant and MDR *Salmonella* spp. increased from 283 and 329 in 1990–1999 (9.52% and 22.94%) to 2510 and 417 in 2000–2009 (84.40% and 29.08%) (Table 2). The majority of AMR *Salmonella* spp. isolates originated from Asia (n = 3579; excluding West Asia). However, an increased number of AMR *Salmonella* spp. originating from West Asia and North Africa (n = 287), and sub-Saharan Africa (n = 96) was reported during 2000–2009. The movements of AMR *Salmonella* spp. can be seen in Figure 3, and Figure 4 shows the movements for typhoidal and non-typhoidal *Salmonella* spp.

**Figure 5:**
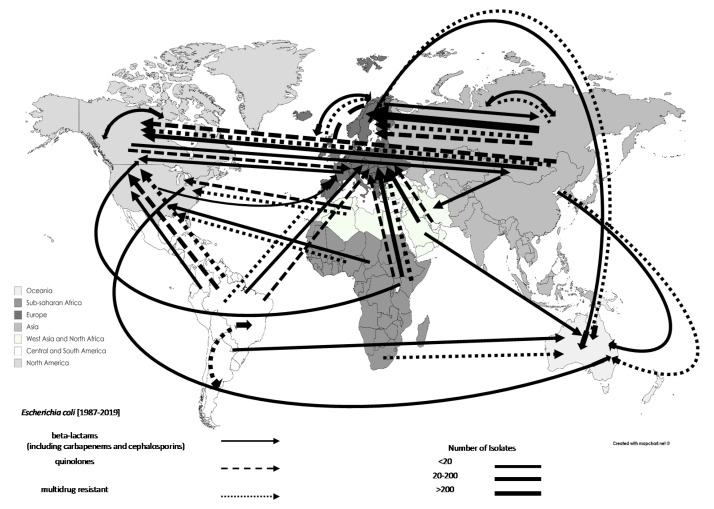
Travel-related antimicrobial resistant *Escherichia coli* transmission, 1987–2019: data are shown by arrows representing antimicrobial resistant isolate movements, where the arrowhead represents the destination and the base of the arrow represents the source. Thus, double-headed arrows represent movements between the same regions. Different regions are represented by different shades.

The total number of AMR *E. coli* isolates associated with travel increased from 591 in 1990–1999 (10.82%) to 3781 in 2000–2009 (69.24%). Specifically, beta-lactam-resistant *E. coli* isolates started to be documented in 2000–2009 (65.52%), with 551 isolates (Table 2). There were documented increases in the rates of reporting for quinolone-resistant and MDR *E. coli* over two decades, increasing from 20 and 36 in 1990–1999 (3.96% and 7.79%) to 380 and 180 in 2000–2009 (75.25% and 38.96%), respectively. However, carbapenem-resistant *E. coli* started to appear in the analysed studies between 2010–2019 (n = 11), with most (72.72%; n = 8) originating from Asia. Overall, AMR *E. coli* mostly originated from Central and South America (n = 1392; 25.49%), and Asia (n = 1169; 21.41%; excluding West Asia). The documented movements of AMR *E. coli* in travellers are displayed in Figure 5.

**Figure 6:**
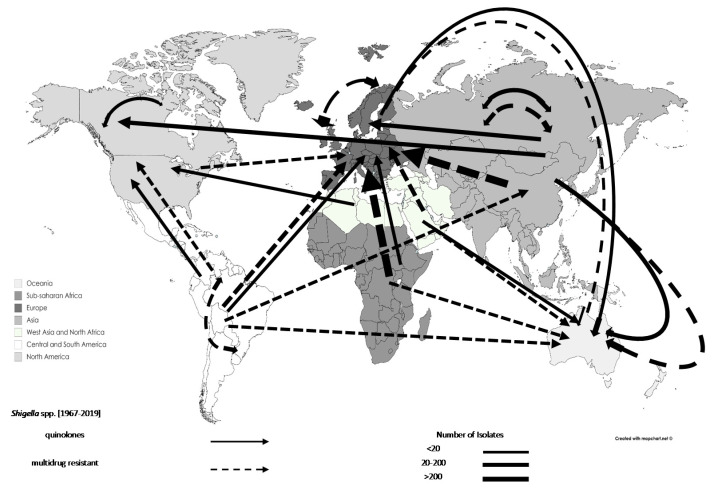
Travel-related antimicrobial resistant *Shigella* spp. movements, 1967–2019: data are shown by arrows representing antimicrobial resistant isolate movements, where the arrowhead represents the destination and the base of the arrow represents the source. Thus, double-headed arrows represent movements between the same regions. Different regions are represented by different shades.

AMR *Shigella* spp. isolates associated with travel increased from 641 before 1990 (9.25%) to 1302 in 1990–1999 (18.79%) and 4526 in 2000–2009 (65.30%). Specifically, MDR *Shigella* spp. increased from 68 before 1990 (4.05%) to 623 in 1990–1999 (37.08%) and 670 in 2000–2009 (39.88%). There was a documented increase of quinolone-resistant *Shigella* spp. from 104 to 183 for the decades 1990–1999 (29.97%) to 2000–2009 (52.74%) (Table 2). Most of the resistant *Shigella* spp. isolates originated from Asia (n = 1697; 24.48%; excluding West Asia), and Central and South America (n = 1242; 17.92%). However, Europe was the source for 100 and 62 MDR *Shigella* spp. during the decades 1990–1999 and 2000–2009, respectively. The movements of AMR *Shigella* spp. can be seen in Figure 6.

The documented isolate numbers for AMR *Campylobacter* spp. isolates that are associated with travel were steady: 1554 in 1990–1999 (47.54%) and 1677 in 2000–2009 (51.30%). However, the rates for quinolone-resistant *Campylobacter* spp. increased from 419 to 1917 between 1990–1999 (16.30%) and 2000–2009 (74.56%) (Table 2). The majority of any AMR- or quinolone-resistant *Campylobacter* spp. originated from Asia (n = 752 or n = 627, 23.00% or 24.39% respectively; excluding West Asia).

Travel-associated AMR *Klebsiella pneumoniae* numbers documented in the literature were low in relation to the corresponding number of studies (n = 187; 27 studies) when compared with other organisms (Table S5). Interestingly, no documentation of AMR *K. pneumoniae* is present before 2000. Resistant *K. pneumoniae* numbers increased in the following two decades: with 25 and 57 MDR isolates detected in 2000–2009 (28.74%) and 2010–2019 (65.52%), respectively, and three and 26 carbapenem-resistant *K. pneumoniae* isolates detected (10.34% and 89.66%), respectively (Table 2). While the countries of origin were not specified for 56.68% (n = 106) of isolates, the most documented source was Asia (except West Asia): n = 31, 16 and 14 for any AMR, MDR and carbapenem-resistant *K. pneumoniae*, respectively. The movements of *K. pneumoniae* isolates associated with travel are illustrated in Figure S1.

### 3.4. AMR Associated with Exposure to Healthcare Systems

The total number of AMR bacteria isolated from medical travellers was 1342 (49 studies). Beta-lactam resistance (including carbapenem and cephalosporin resistance) was identified in 64.01% (n = 859; 24 studies) of AMR bacteria associated with medical travellers. Moreover, 6.33% (n = 85; 5 studies) of bacterial isolates from medical travellers were quinolone-resistant, of which 29.41% of these (n = 24; 3 studies) were beta-lactam co-resistant. Interestingly, MDR organisms comprised 24.14% (n = 324; 32 studies) of medical traveller bacterial isolates. Hence, medical travellers have around twice the odds of detecting MDR bacterial isolates than other travellers (OR = 1.99, *p* < 0.001; considering all isolates are independent observations).

**Figure 7:**
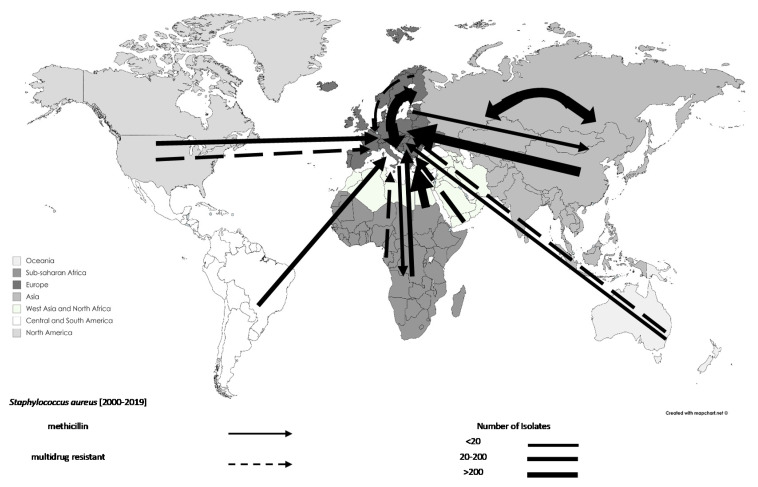
Traveling antimicrobial resistance transmission events for *Staphylococcus aureus*, 2000–2019: data are shown by arrows representing antimicrobial resistant isolate movements, where the arrowhead represents the destination and the base of the arrow represents the source. Thus, double-headed arrows represent movements between the same regions. Different regions are represented by different shades.

AMR *S. aureus* isolates associated with travel increased from 12 in 1990–1999 (0.56%) to 1614 in 2000–2009 (74.65%) and 536 in 2010–2019 (24.79%), with no documented cases before 1990. Interestingly, there were 1781 methicillin-resistant *S. aureus* (MRSA) isolates associated with travel, which started to appear between 2000–2009. Most AMR *S. aureus* isolates originated from Asia (n = 983, 45.47%; excluding West Asia) and Europe (n = 470, 21.74%), of which 77.42% and 95.74% (n = 761 and n = 452, respectively) were MRSA and 4.58% and 0.64% (n = 45 and n = 3, respectively) were MDR, respectively. In addition, 81 MRSA isolates were associated with medical travel, of which 18.52% and 27.16% were from Asia (n = 15; excluding West Asia) and Europe (n = 22), respectively. Interestingly, 18.52% of MRSA cases associated with medical travellers originated from North Africa and West Asia (n = 15). The movements of AMR *S. aureus* can be seen in Figure 7.

Nearly all of the AMR strains of *A. baumannii* (n = 150; 19 studies) and *Pseudomonas aeruginosa* (n = 43; 11 studies) that were reported in the analysed articles were associated with medical travellers; 26.00% (n = 39) of *A. baumannii* strains were categorised as MDR, of which 46.15% (n = 18) showed resistance to the combination of beta-lactams, quinolones and aminoglycosides and 33.33% (n = 50) were categorised as beta-lactam-resistant (all were carbapenem-resistant); and 34.88% (n = 15) of the *P. aeruginosa* strains were categorised as MDR, of which 53.33% (n = 8) showed resistance to the combination of beta-lactams, quinolones and aminoglycosides and 34.88% (n = 15) were categorised as beta-lactam resistant (all were carbapenem- and/or cephalosporin-resistant). Moreover, medical travellers with *P. aeruginosa* infections usually stayed longer in hospitals when they returned, with a mean hospital length of stay of over 45 days [80]. Furthermore, studies documented that *P. aeruginosa* with the *bla*_NDM-1_ resistance gene were first identified in North America and Europe from medical travellers arriving from Asia and Europe, respectively [103,173].

## 4. Discussion

Antimicrobial resistance has become a major global health emergency, with human displacement [271], such as that seen for refugees or travellers, as a facilitative factor [19,20]. While the link between AMR and travel was explored previously, few studies have examined this over time and across different regions of the world [6]. Here, we have compiled and analysed the literature recording the detection of travel-related AMR bacteria during the past three decades.

Prior to 1990, a few studies noted a small number of AMR bacteria being isolated, with the most frequently recorded being *Shigella* spp. from Central and South America or an unspecified location, detected in four studies with 641 isolates. Over time, the frequency of detection of AMR bacteria has increased; however, the number of studies performed has also increased, as has the ease of resistance testing. However, it is clear that there is increasing detection of quinolone-resistant *Campylobacter* spp., MDR *Shigella* spp., quinolone-resistant *Salmonella* spp., and ESBL-producing and quinolone-resistant *E. coli* isolates.

Examining the trends over time and the geographic regions from which AMR appears to be emerging can help inform on how we should be treating travellers returning from these at-risk regions. From our results, we can see that quinolone resistance in *Shigella* spp. was first detected in travellers returning from Oceania before 1990 and Asia between 1990–1999. During the same time period, quinolone-resistant *Salmonella* spp. was also being seen in travellers returning from Asia, with a definite spike in quinolone-resistant and MDR cases of *Salmonella* between 2000 and 2009. While MDR *Shigella* spp. was also seen in travellers returning from Asia in 1990–1999, a number of isolates were also associated with West Asia, and North Africa and Europe, with a more distributed picture occurring between 2000 and 2009.

Gastrointestinal (GI) infections or complaints are commonly associated with travel [272]. Diarrhoea is the most common GI symptom associated with travel and is seen in around 60% of GI cases [273]. Travellers’ diarrhoea occurs less frequently with travel from economically developed countries compared with other countries, and bacteria are the most common microbiologically identified aetiology [273,274]. Unsurprisingly, in our study, enteric bacteria also make up the most frequently occurring AMR species associated with travel. Of particular concern is that AMR enteric bacteria, when acting as either a pathogen or coloniser, can transfer their resistance elements to other commensal or pathogenic bacteria present in the gut [275]. We have found that the number of enteric bacterial isolates that are resistant to antibiotics has been increasing over the years, with quinolone resistance in particular being seen more frequently. The WHO raised international concerns over fluoroquinolone-resistant enteric bacteria in their 2014 report [2]. Also echoed in the WHO report is the growing frequency with which MRSA as well as carbapenemase- and ESBL-producing *Klebsiella pneumoniae* are detected in travellers [6]. As many of the travel-related quinolone-resistant enteric bacteria originated from Asia, we suggest that clinicians who see travellers from this region with GI-related illnesses should avoid empirically prescribing ciprofloxacin or any other quinolones.

The best course of treatment for returning travellers who complain of GI symptoms suggesting a bacterial aetiology should now be considered. Based on practical observations from our data, we would caution against or avoid empirical treatment with beta-lactams and quinolones for bacterial GI travel-related illnesses until an AMR profile or culture and sensitivity testing has been performed. For severe cases with a history of travel to Asia, empirical treatment could start with azithromycin (which was also suggested by other studies [272]) and then change to the drug of choice once the AMR results are back. For travellers returning from other destinations, chloramphenicol could also be used as there are still a low number of documented resistant cases globally (Table S7). For travellers arriving from Africa, Central America or South America, it is advisable to avoid prescribing sulphonamides and trimethoprim due to the high levels of resistance seen in bacteria from these regions. Other general guides for returning travellers with bacterial GI infections include setting and related translational medicine evaluation and implementation [272].

Medical travel-related AMR produces a significant risk that resistance may be introduced to a specific part of a health system or even into a complete health system [276]. Some studies mentioned that medical travel-related AMR isolates cause outbreaks within their receiving institutes [88,268] and suggested protocols for such scenarios [47]. There is a lack of documentation for developing economies’ health systems on how they should prepare when receiving such patients. In addition, monitoring medical-related AMR is challenging, as medical tourism has been increasing in frequency, with associated complications often not being reported as linked to travel [277].

There are a number of limitations with this retrospective systematic review. There is significant variation in the ways in which bacterial species are identified (whether via traditional isolation or PCR) and then undergo antimicrobial susceptibility testing. Often, studies do not clearly link isolates or bacterial species to AMR profiles, and such data are not included as Supplementary Information. Travel histories can be vague and incomplete and are often limited to the destination country because this is the one in which the study is performed, with the origin country being that of most recent travel. This reflects the, often retrospective, nature of the studies and clearly does not have pre- and post-travel samples with a clear itinerary in between, limiting the usefulness and precision of these data. AMR bacteria acquired through travel are also not just a risk for the traveller; some isolates may be detected in family members or other close contacts. Finally, MDR bacteria were properly characterised in the majority of studies (n = 101; 1745 isolates) included in our analysis. In a few studies (n = 16 studies; 2440 isolates), the authors identified MDR bacteria based on the number of clinically relevant antibiotics (at least three) to which these isolates exhibited resistance without specifying these drugs.

A major source of bias in this study is the country in which it was carried out. The majority of cases of travel-related AMR were presented in someone travelling to an economically developing region of the world and then returning to their home country (usually economically developed), being the location where they both fell ill and attended facilities available for testing. The majority of global travel is within-country rather than inter-regional. For example, in 2018, in the USA, domestic air travellers were 3.3-fold more common than international travellers [278]. This means that our findings are necessarily skewed towards developing countries being the origin and developed countries being the destination for AMR bacteria. This is unsurprising as the United Nations Educational, Scientific and Cultural Organization (UNESCO) has found that, between 1996–2015, countries with developed economics spend an average of 1.5% of their GDP on research and development, while countries with economies in transition and countries with developing economies only spend 0.6% of their GDP on research and development [279]. The disproportionate number of studies within different regions therefore limits our understanding of the global picture of travel-related AMR.

## 5. Conclusions

More efforts should focus on the global impacts of travel-related AMR and to encourage studies originating from developing countries. Establishing enteric bacterial AMR profiles for regions with the most traffic to or from may help other healthcare systems address a major part of travel-related AMR. To allow accurate prescription of antimicrobials for patients with complaints that are suspected to be travel-related, hospitalised and inter-healthcare transfers need to be managed in the same way as tertiary-care referrals, with preventive methods on both sides of the journey. Similarly, health authorities may consider the implementation of new guidelines or restrictions for travellers suffering from bacterial infections or those under antibiotic treatment.

## Figures and Tables

**Table 1:** Number of travel-related antimicrobial resistance isolates, documenting studies and year of publication categorised by decade of isolation: isolates that have isolation time frames that overlap decades were categorised into the earliest decade of this time frame.

#	Decade Organism was Isolated		Total Travel-Related AMR Isolates	Total Studies Documenting Travel-Related AMR Isolates ^1^	Year of Publication	
	Start	End			from	to
1	Before 1990		1074	6	1989	1998
2	1990	1999	6570	32	1993	2019
3	2000	2009	16,688	91	2001	2019
4	2010	2019	5003	126	2010	2019
Total	Until Jun 2019 (inclusive)		30,060 ^2^	238 ^2^	1989	2019

^1 ^ Some studies may document multiple isolates from different decades. Therefore, they may not add up to the total given. ^2 ^ Includes 725 isolates with a broad isolation time frame, 1984–2015, from one study; AMR, antimicrobial resistant.

**Table 2:** Distribution of the number of AMR isolates by decade for nominated species ^1^ separated by source.

Decade		Before 1990				1990–1999				2000–2009				2010–2019				Total			
Organism	Region	BL/CP	QR	MDR	All AMR	BL/CP	QR	MDR	All AMR	BL/CP	QR	MDR	All AMR	BL/CP	QR	MDR	All AMR	BL/CP	QR	MDR	All AMR
*Klebsiella pneumoniae*	Sub-Saharan Africa	0/0	0	0	0	0/0	0	0	0	0/0	0	0	0	4/2	0	1	5	4/2	0	1	5
	North Africa and West Asia	0/0	0	0	0	0/0	0	0	0	0/0	0	2	2	7/1	0	10	19	7/1	0	12	21
	Asia (except West Asia)	0/0	0	0	0	0/0	0	0	0	2/2	0	1	3	12/11	0	15	28	14/13	0	16	31
	Europe	0/0	0	0	0	0/0	0	0	0	0/0	0	6	6	2/0	0	11	13	2/0	0	17	19
	North America	0/0	0	0	0	0/0	0	0	0	0/0	0	0	0	1/0	0	2	3	1/0	0	2	3
	South and Central America	0/0	0	0	0	0/0	0	0	0	0/0	0	0	0	1/0	0	1	2	1/0	0	1	2
	Oceania	0/0	0	0	0	0/0	0	0	0	0/0	0	0	0	0/0	0	0	0	0/0	0	0	0
	Other	0/0	0	0	0	0/0	0	0	0	1/1	0	16	61	12/12	12	21	45	13/13	12	37	106
*Campylobacter* spp.	Sub-Saharan Africa	0/0	0	0	0	0/0	52	0	98	0/0	99	0	140	0/0	0	0	0	0/0	151	0	238
	North Africa and West Asia	0/0	0	0	0	0/0	8	0	8	0/0	67	0	90	0/0	0	0	0	0/0	75	0	98
	Asia (except West Asia)	0/0	0	0	0	0/0	227	0	232	0/0	383	1	502	0/0	17	1	18	0/0	627	2	752
	Europe	0/0	0	0	0	0/0	90	0	90	0/0	376	0	402	0/0	0	1	1	0/0	466	1	493
	North America	0/0	0	0	0	0/0	0	0	0	0/0	3	0	4	0/0	0	0	0	0/0	3	0	4
	South and Central America	0/0	0	0	0	0/0	42	0	76	0/0	274	0	299	0/0	0	0	0	0/0	316	0	375
	Oceania	0/0	0	0	0	0/0	0	0	0	0/0	0	0	0	0/0	0	0	0	0/0	0	0	0
	Other	0/0	0	0	19	0/0	0	0	1050	0/0	715	0	240	0/0	218	0	0	0/0	933	0	1309
*Shigella* spp.	Sub-Saharan Africa	0/0	0	0	0	0/0	4	187	337	0/0	1	181	426	0/0	0	34	42	0/0	5	402	805
	North Africa and West Asia	0/0	0	0	0	0/0	0	1	60	0/0	3	8	66	1/0	0	48	70	1/0	3	57	196
	Asia (except West Asia)	0/0	0	0	0	0/0	99	251	434	0/0	143	262	1023	8/0	48	127	240	8/0	280	640	1697
	Europe	0/0	0	0	0	0/0	0	100	100	0/0	0	62	67	0/0	1	3	10	0/0	1	165	177
	North America	0/0	0	0	0	0/0	0	0	0	0/0	6	0	180	0/0	0	3	4	0/0	6	3	184
	South and Central America	0/0	0	31	150	0/0	1	57	201	0/0	8	53	824	2/0	2	28	67	2/0	11	169	1242
	Oceania	0/0	0	0	0	0/0	0	0	0	0/0	0	0	11	1/0	0	0	9	1/0	0	4	20
	Other	0/0	18	37	491	0/0	0	27	170	0/0	22	104	1929	1/0	1	11	20	1/0	41	179	2610
*Salmonella* spp.	Sub-Saharan Africa	0/0	0	0	0	0/0	4	3	14	0/0	39	27	96	0/0	1	3	5	0/0	44	33	115
	North Africa and West Asia	0/0	0	0	0	0/0	23	1	25	0/0	282	1	287	0/0	9	8	20	0/0	314	10	332
	Asia (except West Asia)	0/0	0	16	16	0/0	220	154	403	0/0	1905	354	2344	0/0	50	35	91	0/0	2290 ^2^	1169 ^3^	3579 ^2,3^
	Europe	0/0	0	0	0	0/0	24	2	332	0/0	70	0	74	0/0	4	0	4	0/0	98	2	410
	North America	0/0	0	0	0	0/0	0	0	0	0/0	0	0	2	0/0	0	0	0	0/0	0	0	2
	South and Central America	0/0	0	1	1	0/0	3	2	6	0/0	14	5	35	0/0	1	14	15	0/0	18	22	57
	Oceania	0/0	0	0	0	0/0	0	0	0	0/0	0	0	1	0/0	0	0	0	0/0	0	0	1
	Other	0/0	0	0	51	0/0	9	167	773	0/0	200	30	710	0/0	1	1	2	0/0	210	198	1536
*Escherichia coli*	Sub-Saharan Africa	0/0	0	0	0	0/0	0	4	5	46/0	0	9	55	17/0	1	29	49	63/0	1	42	109
	North Africa and West Asia	0/0	0	0	0	0/0	0	13	14	70/0	15	9	178	3/0	23	3	34	73/0	38	25	226
	Asia (except West Asia)	0/0	0	0	0	0/0	6	7	14	352/0	134	41	950	106/8	16	68	205	458/8	156	116	1169
	Europe	0/0	0	0	0	0/0	0	0	0	19/0	0	0	24	71/1	23	4	108	90/1	23	4	132
	North America	0/0	0	0	25	0/0	0	0	0	35/0	0	0	38	4/0	3	0	7	39/0	3	0	70
	South and Central America	0/0	0	25	203	0/0	1	12	75	28/0	171	13	1090	5/0	7	12	24	33/0	179	62	1392
	Oceania	0/0	0	0	0	0/0	0	0	0	0/0	0	0	0	0/0	0	0	0	0/0	0	0	0
	Other	0/0	0	0	88	0/0	13	0	483	1/0	60	108	1446	84/2	32	101	346	85/2	105	209	2363
*Staphylococcus aureus*		MRSA				MRSA				MRSA				MRSA				MRSA			
	Sub-Saharan Africa	0	0	0	0	0	0	0	0	57	0	1	58	7	2	2	118	64	2	3	176
	North Africa and West Asia	0	0	0	0	0	0	0	0	139	0	0	141	16	0	0	16	155	0	0	157
	Asia (except West Asia)	0	0	0	0	0	0	0	0	705	0	31	737	56	20	23	201	761	20	54	938
	Europe	0	0	0	0	0	0	0	0	426	0	1	431	26	0	2	39	452	0	3	470
	North America	0	0	0	0	0	0	0	0	84	0	1	90	5	0	0	5	89	0	1	95
	South and Central America	0	0	0	0	0	0	0	0	87	0	0	88	30	3	0	57	117	3	0	145
	Oceania	0	0	0	0	0	0	0	0	25	0	1	27	1	1	0	6	26	1	1	33
	Other	0	0	0	0	0	0	9	12	39	1	1	42	78	14	16	94	117	15	26	148

^1^ Nominated organisms (*Campylobacter* spp., *Escherichia coli*, *Klebsiella pneumoniae*, *Salmonella* spp., *Shigella* spp. and *Staphylococcus aureus*). ^2^ Includes 115 isolates with a broad isolation time frame, 1984–2015. ^3^ Includes 610 isolates with a broad isolation time frame, 1984–2015; AMR: antimicrobial resistance; All AMR: the total number of isolates with any reported AMR; CP: carbapenem resistance, not exclusive from beta-lactam resistance; BL: beta-lactam resistance, includes carbapenem and/or cephalosporin resistance; MDR: multidrug-resistant organisms, organisms documented as multidrug-resistant, or resistant to three or more classes of antimicrobials; MRSA: methicillin-resistant *Staphylococcus aureus*; QR: quinolone resistant, may include co-resistance with beta-lactams or methicillin; Other: unspecified or multiple regions were documented.

## Data Availability

Not applicable.

## Supplementary Materials

The following are available online at https://www.mdpi.com/2414-6366/6/1/11/s1, Figure S1. Travel-related antimicrobial resistant Klebsiella pneumoniae movements, 2000–2019, Table S1. List of the included studies in the review, Table S2. List of Countries or regions that are documented in the review, Table S3. Top originating regions for AMR organisms (source), Table S4. Top destination regions for AMR organisms (destination), Table S5. Number of studies and isolates for species that were documented in the analyzed studies, Table S6. Numbers of travel-related AMR isolates documented in 238 studies, up to June 2019 inclusive, categorized by source and isolation time, Table S7. Number of travel-related isolates for enteric organisms with documented AMR component, Table S8. Antimicrobials categories that were included in the analysis.
