# Supplementary material for: Travel-Related Antimicrobial Resistance: A Systematic Review
Source: Trop Med Infect Dis. 2021 Jan 16;6(1):11. doi: 10.3390/tropicalmed6010011 (PMC7838817; doi:10.3390/tropicalmed6010011)
Supplement: Supplementary file 1 [file tropicalmed-06-00011-s001.pdf]

Tropical Medicine and Infectious Disease

MDPI

Supplementary Materials

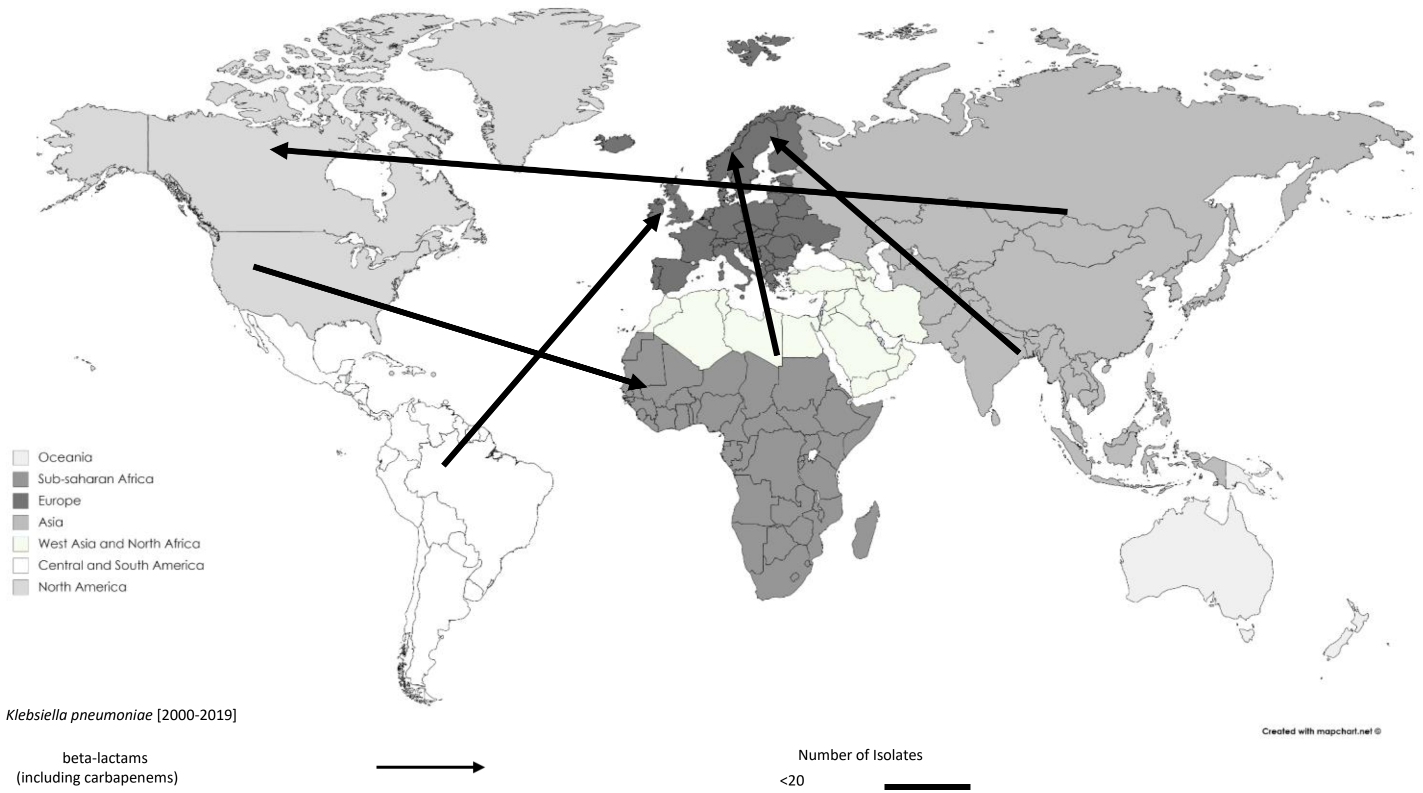

Figure S1. Travel-related antimicrobial resistant *Klebsiella pneumoniae* movements, 2000–2019. Data shown by arrows representing antimicrobial resistant (AMR) isolate movements, where the arrowhead represents the destination and the base of the arrow represents the source. Thus, double headed arrows represent movements between the same regions. Different regions are represented with different shades.

Table S1. List of the included studies in the review.

Publication year	First Author's last name	Title	Listed reduced sensitivity species	Reported reduced sensitivity or resistant antimicrobials	Travelling from	Travelling to	Assessment Score #
1989	Parsonnet [194]	Shigella dysenteriae type 1 infections in US travellers to Mexico, 1988	Shigella dysenteriae	tetracycline, sulfafurazole, streptomycin, trimethoprim, TMP-SMZ, ampicillin and chloramphenicol	Mexico	USA	C
1990	Murray [177] +,*	Emergence of resistant faecal Escherichia coli in travellers not taking prophylactic antimicrobial agents	Escherichia coli	trimethoprim, ampicillin, chloramphenicol, gentamicin, kanamycin, streptomycin, sulfonamides, tetracycline and TMP-SMZ	USA and Mexico	Mexico and USA	B
1990	Tauxe [238]	Antimicrobial resistance of Shigella isolates in the USA: The importance of international travelers	Shigella spp.	ampicillin, chloramphenicol, streptomycin, sulfisoxazole, tetracycline and TMP-SMZ	Unspecified	USA	A
1993	Bourgeois [59]	Etiology of acute diarrhea among United States military personnel deployed to South America and west Africa	Escherichia coli, Salmonella spp., Shigella spp., Campylobacter spp., Aeromonas spp., Plesiomonas spp. and Vibrio spp.	ampicillin, TMP-SMZ and tetracycline	West Africa and South America	USA	C

1993	Cohen [72]	Colonization by enteroaggregative Escherichia coli in travelers with and without diarrhea	Escherichia coli	TMP-SMZ	Central America, South America, The Caribbean or Mexico	USA	A
1994	Oh [186]	Multidrug-resistant Typhoid Fever in Singapore	Salmonella enterica serotype Typhi	chloramphenicol, ampicillin and TMP-SMZ	Bangladesh and India	Singapore	B
1994	Vila [246]	Antimicrobial resistance of Shigella isolates causing Traveller's Diarrhea	Shigella sonnei and Shigella flexneri	ampicillin, chloramphenicol, TMP-SMZ, tetracycline and cephalothin	East Africa, North Africa, West Africa, East Asia, Southeast Asia, South Asia, South America and Central America	Spain	B
1997	Ohtaka [187]	Epidemiological approach to the prevention of imported infectious diseases in the age of globalization	Shigella (flexneri, boydii, sonnei and dysenteriae) and Vibrio cholera	ampicillin, streptomycin, erythromycin, tetracycline, ciprofloxacin and TMP-SMZ	India, Thailand and unspecified	Japan	C
1998	Harnett [106]	Molecular characterization of multiresistant strains of Salmonella typhi from South Asia isolated in Ontario, Canada	Salmonella enterica serotype Paratyphi A and Typhi	ampicillin, chloramphenicol, tetracycline, trimethoprim, ticarcillin, piperacillin, TMP-SMZ, streptomycin, sulfamethoxazole, cephalothin, nitrofurantoin and nalidixic acid	Bangladesh, India, Pakistan and Sri Lanka	Canada	C
1998	Mermin [170]	Typhoid Fever in the United States, 1985-1994: changing risks of international travel and increasing antimicrobial resistance	Salmonella enterica serotype Typhi	ampicillin, chloramphenicol and TMP-SMZ	Bangladesh, India, Myanmar, Pakistan, Sri Lanka and Central America	USA	B

2000	Ackers [33]	Laboratory-based surveillance of Salmonella serotype Typhi infections in the United States	Salmonella enterica serotype Typhi	nalidixic acid, ampicillin, chloramphenicol streptomycin, tetracycline, sulfafurazole and TMP-SMZ	Bangladesh, Haiti, India, Netherlands, Pakistan, Philippines, Turkey, UK and Vietnam	USA	B
2000	Daniels [75] *	Traveler's diarrhea at sea: Three outbreaks of waterborne enterotoxigenic Escherichia coli on cruise ships	Escherichia coli	ampicillin, amoxicillin/clavulanic acid, chloramphenicol, streptomycin, sulfafurazole, TMP-SMZ and tetracycline	Jamaica and Mexico	USA and Jamaica	C
2000	Vila [248]	Quinolone resistance in enterotoxigenic Escherichia coli causing diarrhea in travelers to India in comparison with other geographical areas	Escherichia coli	ampicillin, amoxicillin/clavulanic acid, chloramphenicol, ciprofloxacin, nalidixic acid, tetracycline and TMP-SMZ	India and unspecified	Spain	C
2001	Hakanen [103]	Reduced Fluoroquinolone susceptibility in Salmonella enterica serotypes in travelers returning from Southeast Asia	Salmonella enterica spp. (Including serotypes Enteritidis, Typhimurium, Hadar, Virchow, Newport, Infantis, Anatum and Panama)	ciprofloxacin, tetracycline, sulfamethoxazole, ampicillin, trimethoprim streptomycin and chloramphenicol	Cyprus, Dominican Republic, Estonia, Greece, India, Indonesia, Israel, Kenya, Malaysia, Morocco, Russia, Spain, Sri Lanka, Thailand, Tunisia, Turkey and unspecified	Finland	B
2001	Huang [117] *	Emergence of trimethoprim-resistant Escherichia coli in healthy persons in the absence of prophylactic or therapeutic antibiotics during travel to Guadalajara, Mexico	Escherichia coli	ampicillin, azithromycin, chloramphenicol, ciprofloxacin, doxycycline, erythromycin, furazolidone, levofloxacin, trimethoprim, TMP-SMZ and trovafloxacin	Mexico	USA	C

2001	Vila [249]	Susceptibility patterns of enteroaggregative Escherichia coli associated with travellers' diarrhoea: Emergence of quinolone resistance	Escherichia coli	nalidixic acid, ampicillin, chloramphenicol, tetracycline, TMP-SMZ and ciprofloxacin	India and Central America	Spain	C
2002	The Campylobacter Sentinel Surveillance Scheme Collaborators [65]	Ciprofloxacin resistance in Campylobacter jejuni: Case–case analysis as a tool for elucidating risks at home and abroad	Campylobacter jejuni	ciprofloxacin	Cyprus, France, Kenya, Mauritius, Morocco, Portugal, South Africa, Spain, Tanzania, Tunisia, Turkey and unspecified	UK	B
2002	Jiang [130] *	Prevalence of enteric pathogens among international travelers with diarrhea acquired in Kenya (Mombasa), India (Goa), or Jamaica (Montego Bay)	Aeromonas spp., Campylobacter spp., Escherichia coli, Plesiomonas spp., Salmonella spp., Shigella spp. and Vibrio spp.	ampicillin, chloramphenicol, doxycycline, furazolidone, gentamycin, ofloxacin, streptomycin, sulfisoxazole, tetracycline, trimethoprim and TMP-SMZ	India, Jamaica and Kenya	Not mentioned	B
2003	Hakanen [102]	Fluoroquinolone resistance in Campylobacter jejuni isolates in travelers returning to Finland: Association of ciprofloxacin resistance to travel destination	Campylobacter jejuni	ciprofloxacin	Australia, India, Portugal, Spain, Thailand, Tunisia, Turkey, Asia, Africa, Europe and America	Finland	C
2003	Vila [247]	Aeromonas spp. and Traveler's Diarrhea: Clinical features and antimicrobial resistance	Aeromonas caviae and veronica biotype sorbia	ampicillin, cefotaxime, chloramphenicol, ciprofloxacin, nalidixic acid, tetracycline and TMP-SMZ	Burkina Faso, Guatemala, India, Iran, Kenya, Mali, Mexico, Nepal, Nicaragua, Paraguay, Senegal, Thailand and Sub-Saharan Africa	Spain	C

2004	Cabrera [63]	Mechanism of resistance to several antimicrobial agents in Salmonella Clinical isolates causing traveler's diarrhea	Salmonella enterica spp. (Including serotypes Enteritidis, Goldcoast, Hadar, Haifa, Kiambu, Paratyphi, Risseu, Typhimurium and Virchow)	ampicillin, amoxicillin/clavulanic acid, nalidixic acid, gentamicin, TMP-SMZ, tetracycline and chloramphenicol	Bolivia, Egypt, Gambia, India, Ivory Coast, Kenya, Mali, Mexico, Morocco, Peru, Senegal and unspecified	Spain	C
2004	Fischer [91]	Bacterial colonization of patients undergoing international air transport: A prospective epidemiologic Study	Staphylococcus aureus, Acinetobacter baumannii and Klebsiella pneumoniae	methicillin and MDR	Germany, Italy, Spain and Romania	Germany	C
2004	Iverson [126]	Fluoroquinolone resistance among Neisseria gonorrhoeae isolates in Hawaii, 1990-2000: Role of foreign importation and increasing endemic spread	Neisseria gonorrhoeae	ciprofloxacin	Asia or Oceania	USA	B
2004	Kassenborg [138]	Fluoroquinolone-resistant Campylobacter infections: Eating poultry outside of the home and foreign travel are risk factors	Campylobacter spp.	Fluoroquinolones	Mexico, Asia, Central America, South America and Western Europe	USA	B

2004	Navia [180]	Molecular characterization of the integrons in Shigella strains isolated from patients with traveler's diarrhea	Shigella flexneri, Shigella sonnei and Shigella dysenteriae	ampicillin, tetracycline, chloramphenicol, TMP-SMZ and trimethoprim	Egypt, Guatemala, Kenya, Mexico, Nicaragua, Tunisia, Turkey, Venezuela and Sub-Saharan Africa	Spain	C
2005	Aardema [32]	Changing epidemiology of melioidosis? A case of acute pulmonary melioidosis with fatal outcome imported from Brazil	Burkholderia pseudomallei	gentamicin, cefuroxime and erythromycin	Brazil	Netherlands	A
2005	Alcoba-Florez [40] *	Outbreak of Shigella sonnei in a rural hotel in La Gomera, Canary Islands, Spain	Shigella sonnei	amikacin, cefaclor, cefalotin, cefuroxime, cefuroxime axetil, gentamicin, nitrofurantoin, tobramycin and TMP-SMZ	Spain **	Not mentioned	B
2005	Denis [80]	Polyclonal emergence and importation of community-acquired methicillin-resistant Staphylococcus aureus strains harbouring Panton-Valentine leucocidin genes in Belgium	Staphylococcus aureus	methicillin, penicillin and oxacillin	North Africa and Ecuador	Belgium	C

2005	Kubota [148]	Analysis of Salmonella enterica serotype Typhi pulsed-field gel electrophoresis patterns associated with international travel	Salmonella enterica serotype Typhi	ampicillin, chloramphenicol, nalidixic acid, TMP-SMZ, sulfasoxazole, streptomycin and tetracycline	Bangladesh, Haiti, India, Pakistan and Vietnam	USA	C
2005	Maier [162]	Panton-Valentine leukocidin-positive methicillin-resistant Staphylococcus aureus in Germany associated with travel or foreign family origin	Staphylococcus aureus	erythromycin, fusidic acid, methicillin and oxacillin	Germany, Saudi Arabia, Pakistan and USA	Egypt, Germany, Philippines, UK and West Asia	C
2005	Navia [179]	Analysis of mechanisms of resistance to several antimicrobial agents in Shigella spp. causing travellers diarrhoea	Shigella flexneri and Shigella sonnei	ampicillin, tetracycline, chloramphenicol and trimethoprim	India, Nepal, North Africa, Sub-Saharan Africa, Central America, South America, Southeast Asia, West Asia and unspecified	Spain	C
2005	Olsen [188]	VTEC O117:K1:H7 A new clinal group of E. coli associated with persistent diarrhoea in Danish travellers	Escherichia coli	streptomycin, sulfonamides and trimethoprim	Bangladesh, Cuba, Egypt, Eritrea, Ghana, India, Iraq, Iran, Israel, Malaysia, Pakistan, Tanzania and Thailand	Denmark	C
2005	Shrestha [224]	Community-acquired methicillin-resistant Staphylococcus aureus in a returned traveler	Staphylococcus aureus	methicillin	Democratic Republic of the Congo	USA	A
2006	Weyrich [255]	Fatal multidrug-resistant Acinetobacter baumannii sepsis in a patient with travel history and recent onset of systemic lupus erythematosus: A case report	Acinetobacter baumannii	penicillins, cephalosporins, aminoglycosides, fluoroquinolones, rifampicin, tetracyclines, oxazolidinones and macrolides	Greece	Germany	B

2007	Ruiz [211]	Trends in antimicrobial resistance in Campylobacter spp. causing traveler's diarrhea	Campylobacter spp.	amoxicillin/clavulanic acid, ampicillin, tetracycline, nalidixic acid, ciprofloxacin, chloramphenicol and clindamycin	Indonesia, Nicaragua, Peru, Africa, America and Asia	Spain	C
2007	Wybo [259]	Outbreak of multidrug-resistant Acinetobacter baumannii in a Belgian university hospital after transfer of patients from Greece	Acinetobacter baumannii	carbapenems, beta-lactams, fluoroquinolones and amikacin	Greece	Belgium	A
2008	Al Naiemi [39]	Extended-spectrum-beta-lactamase production in a Salmonella enterica serotype Typhi strain from the Philippines	Salmonella enterica serotype Typhi	ampicillin, ceftazidime, cefotaxime, cefpodoxime, ciprofloxacin, gentamicin, tobramycin, piperacillin and TMP-SMZ	Philippines	Netherlands	C
2008	Arai [47]	Epidemiological evidence of multidrug-resistant Shigella sonnei colonization in India by sentinel surveillance in a Japanese quarantine station	Shigella sonnei	tetracycline, TMP-SMZ and nalidixic acid	India	Japan	C
2008	Bochet [56]	Community-acquired methicillin-resistant Staphylococcus aureus infections in two scuba divers returning from the Philippines	Staphylococcus aureus	methicillin and beta-lactams	Philippines	Switzerland	B
2008	Gupta [101]	Laboratory-based surveillance of Paratyphoid Fever in the United States: Travel and antimicrobial resistance	Salmonella enterica serotype Paratyphi A	nalidixic acid, ciprofloxacin and multidrug resistant	Bangladesh, India, Indonesia and Pakistan	USA	C

2008	Haukka [111]	Emerging resistance to newer antimicrobial agents among Shigella Isolated from Finnish foreign travellers	Shigella spp.	ampicillin, chloramphenicol, streptomycin, sulfonamide, gentamicin, cefoxime, tetracycline, trimethoprim, ciprofloxacin, Nalidixic acid, mecillinam, imipenem, neomycin, cefotaxime and multidrug resistant	China, Egypt, Asia, Africa, Americas and Europe	Finland	B
2008	Larsen [152]	Epidemiology of European community-associated methicillin-resistant Staphylococcus aureus clonal complex 80 type IV strains isolated in Denmark from 1993 to 2004	Staphylococcus aureus	streptomycin, tetracycline, kanamycin, erythromycin, clindamycin and fusidic acid	Unspecified	Denmark	C
2008	Laupland [153]	Community-onset extended-spectrum beta-lactamase (ESBL) producing Escherichia coli: Importance of international travel	Escherichia coli	ciprofloxacin, gentamicin, nitrofurantoin and TMP-SMZ	India, Mexico, North Africa, Sub-Saharan Africa, West Asia and Asia	Canada	C
2008	Mensa [169]	Quinolone resistance among Shigella spp. isolated from travellers returning from India	Shigella spp.	ciprofloxacin, quinolone and nalidixic acid	India, Equatorial Guinea and Mali	Spain	C
2008	Pontali [201]	Imported typhoid fever with hepatitis from Bangladesh: A case of delayed response to ceftriaxone?	Salmonella enterica serotype Typhi	ampicillin, cefazolin, cefoxitin, chloramphenicol, ciprofloxacin, gentamicin, levofloxacin and TMP-SMZ	Bangladesh	Italy	B

2008	Schleucher [218]	Panton-Valentine leukocidin-producing methicillin-sensitive Staphylococcus aureus as a cause for recurrent, contagious skin infections in young, healthy travelers returned from a tropical country: A new worldwide public health problem?	Staphylococcus aureus	Penicillin G	Fiji	Germany	B
2008	Szabo [231]	Imported PER-1 producing Pseudomonas aeruginosa, PER-1 producing Acinetobacter baumanii and VIM-2-producing Pseudomonas aeruginosa strains in Hungary	Pseudomonas aeruginosa, Acinetobacter baumannii and Klebsiella pneumoniae	amikacin, aztreonam, cefepime, cefotaxime, ceftazidime, ceftriaxone, ciprofloxacin, gentamicin, imipenem, imipenem + EDTA, meropenem, netilmicin, piperacillin/tazobactam and tobramycin	Egypt	Hungary	A
2008	Vlieghe [250]	Trends of norfloxacin and erythromycin resistance of Campylobacter jejuni/Campylobacter coli isolates recovered from international travelers, 1994 to 2006	Campylobacter jejuni and/or Campylobacter coli	erythromycin and norfloxacin	Egypt, Haiti, India, Iran, Lebanon, Spain, Thailand, North Africa, Sub-Saharan Africa, Central America, South America, West Asia, East Asia, South Asia	Belgium	B
2009	Cabrera [64]	Characterization of the enzyme aac(3)-id in a clinical isolate of Salmonella enterica serovar Haifa causing traveler's diarrhea	Salmonella enterica serotype Haifa	gentamicin, nalidixic acid and tetracycline	Egypt	Spain	C

2009	Evans [88]	Risk factors for ciprofloxacin-resistant Campylobacter infection in Wales	Campylobacter spp.	ciprofloxacin	Spain and unspecified	UK	B
2009	Hume [120]	Increasing rates and clinical consequences of nalidixic acid-resistant isolates causing enteric fever in returned travellers: an 18-year experience	Salmonella enterica serotype Typhi and Salmonella enterica serotype Paratyphi	nalidixic acid	Bangladesh, Cambodia, China, India, Malaysia, Pakistan, and Sri Lanka	Australia	C
2009	Izumiya [127]	Characterization of Shigella sonnei isolates from travel-associated cases in Japan	Shigella sonnei	nalidixic acid	China, India, Korea, Nepal, Thailand, Peru and Vietnam	Japan	C
2009	Lindgren [158]	Reduced fluoroquinolone susceptibility in Salmonella enterica isolates from Travelers, Finland	Salmonella enterica spp.	ciprofloxacin and nalidixic acid	Egypt, India, Malaysia, Morocco, Portugal, Spain, Tanzania, Thailand and Vietnam	Finland	C
2009	Mendez Arancibia [168]	Evolution of antimicrobial resistance in enteroaggregative Escherichia coli and enterotoxigenic Escherichia coli causing traveller's diarrhoea	Escherichia coli	ampicillin, amoxicillin/clavulanic acid, chloramphenicol, tetracycline, nalidixic acid, ciprofloxacin and TMP-SMZ	Unspecified	Spain	C
2009	Pitout [198]	Molecular characteristics of travel-related extended-spectrum-β-lactamase-producing Escherichia coli isolates from the Calgary Health Region	Escherichia coli	beta-lactams, TMP-SMZ, piperacillin/tazobactam, amoxicillin/clavulanic acid, tobramycin, gentamicin, amikacin, nitrofurantoin, and ciprofloxacin.	USA, Sub-Saharan Africa, North Africa, South America, Central America, West Asia, South Asia, Asia and Europe	Canada	C
2009	Taguchi [232]	Plasmid-mediated quinolone resistance in Salmonella isolated from patients with overseas Travelers' Diarrhea in Japan	Salmonella enterica spp.	ampicillin, streptomycin, tetracycline, chloramphenicol, TMP-SMZ and nalidixic acid	Indonesia, Malaysia, Singapore, Thailand and Vietnam	Japan	C

2009	Yong [261]	Characterization of a new metallo-β-lactamase gene, blaNDM-1, and a novel erythromycin esterase gene carried on a unique genetic structure in Klebsiella pneumoniae sequence type 14 from India	Klebsiella pneumoniae	ampicillin, piperacillin, cephalothin, cefoxitin, cefotaxime, cefuroxime, ceftazidime, aztreonam, cefepime, ertapenem, imipenem, meropenem and ciprofloxacin	India	Sweden	C
2010	Drews [82]	Laboratory based surveillance of travel-related Shigella sonnei and Shigella flexneri in Alberta from 2002 to 2007	Shigella flexneri and Shigella sonnei	ampicillin, amoxicillin/clavulanic acid, ceftiofur, ceftriaxone, TMP-SMZ, sulfisoxazole, chloramphenicol, ciprofloxacin, nalidixic acid, gentamicin, streptomycin and tetracycline	North Africa, Sub-Saharan Africa, North America, Central America, South America, West Asia, South Asia and East Asia	Canada	C
2010	Ellington [83]	First international spread and dissemination of the virulent Queensland community-associated methicillin-resistant Staphylococcus aureus strain	Staphylococcus aureus	methicillin, oxacillin, erythromycin and rifampicin	Australia	UK	C
2010	Kennedy [24] +	Colonisation with Escherichia coli resistant to "critically important" antibiotics: a high risk for international travellers	Escherichia coli, Klebsiella spp. and Morganella spp.	gentamicin, ciprofloxacin, beta-lactams and cephalosporins (3rd Generation)	China, Hong Kong, India, Korea, Mexico, Nepal, Sri Lanka, Taiwan, North Africa, Sub-Saharan Africa, North America, South America, Southeast Asia, West Asia, Europe and Oceania	Australia	B

2010	Kishore [141]	Fever and abdominal pain in a returning traveller	Salmonella enterica serotype Typhi	amoxicillin and chloramphenicol	Pakistan	UK	C
2010	Patel [195]	Imported enteric fever: Case series from the hospital for tropical diseases, London, United Kingdom	Salmonella enterica serotype Typhi and Salmonella enterica serotype Paratyphi	ciprofloxacin, ampicillin, chloramphenicol, fluoroquinolone, nalidixic acid and TMP-SMZ	Nigeria and South Asia	UK	C
2010	Porter [202]	The epidemiology of travelers' diarrhea in Incirlik, Turkey: A region with a predominance of heat-stabile toxin producing enterotoxigenic Escherichia coli	Escherichia coli and Campylobacter spp.	ciprofloxacin, TMP-SMZ, tetracycline and nalidixic acid	Turkey	USA	C
2010	Sharafeldin [221]	Health risks encountered by Dutch medical students during an elective in the tropics and the quality and comprehensiveness of pre-and post-travel care	Staphylococcus aureus	methicillin	Unspecified	Netherlands	C
							A
2010	Stenhem [229]	Imported methicillin-resistant Staphylococcus aureus, Sweden	Staphylococcus aureus	methicillin	Cyprus, Greece, Ireland, Lebanon, Spain, Syria, Thailand, Turkey, UK, USA, Yugoslavia, North Africa, Sub-Saharan Africa, North America, South America, East Asia, West Asia, Europe and Oceania	Sweden	C

2010	Tangden [234]	Foreign travel is a major risk factor for colonization with Escherichia coli producing CTX-M-type extended-spectrum β-lactamases: a prospective study with Swedish volunteers	Escherichia coli	beta-lactams	India, Sub-Saharan Africa, North Africa, West Asia, Asia and Europe	Sweden	C
2010	Tappe [235]	Panton-Valentine leukocidin-positive Staphylococcus aureus infections in returning travelers	Staphylococcus aureus	methicillin	Costa Rica, Pakistan and Thailand	Germany	B
2011	Al-Mashhadani [45]	Foreign travel and decreased ciprofloxacin susceptibility in Salmonella enterica infections	Salmonella enterica serotypes Enteritidis, Typhimurium, Newport, Virchow and other	nalidixic acid	Unspecified	UK	B
2011	Bottieau [58]	Epidemiology and outcome of Shigella, Salmonella and Campylobacter infections in travellers returning from the tropics with fever and diarrhoea	Shigella spp., Salmonella spp. and Campylobacter jejuni	ampicillin, norfloxacin and TMP-SMZ	Sub-Saharan Africa, North Africa, South America, South Asia and West Asia	Belgium	B
2011	Chan [67]	The perils of medical tourism: NDM-1-positive Escherichia coli causing febrile neutropenia in a medical tourist	Escherichia coli	amikacin, ampicillin, ceftriaxone, ceftazidime, ciprofloxacin, ertapenem, gentamicin, imipenem, meropenem and TMP-SMZ	Bangladesh	Singapore	C

2011	Christenson [70]	Methicillin-resistant Staphylococcus aureus infection in Uppsala County, Sweden	Staphylococcus aureus	clindamycin, fusidic acid, isoxapenicillin and tetramycin	Brazil, Egypt, Greece, India, Italy, Japan, Norway, UK, USA, North Africa and West Asia	Sweden	C
2011	Dhanji [26]	Variation in the genetic environments of blaCTX-M-15 in Escherichia coli from the faeces of travellers returning to the United Kingdom	Escherichia coli	cefotaxime, ceftazidime, piperacillin, piperacillin/tazobactam, ciprofloxacin, amikacin, gentamicin, tobramycin and beta-lactams	Afghanistan, Egypt, India, Kenya, Kuwait, Pakistan, Thailand	UK	B
2011	Gilmour [98]	Vibrio cholerae in traveler from Haiti to Canada	Vibrio cholerae	TMP-SMZ	Haiti	Canada	C
2011	Guiral [99]	CTX-M-15–producing enteroaggregative Escherichia coli as cause of travelers' diarrhea	Escherichia coli	ampicillin, piperacillin, amoxicillin/clavulanic acid, ampicillin/sulbactam, cefuroxime, Cefazoline, cefepime, cefotaxime, ceftazidime, gentamicin, tobramycin, aztreonam, ciprofloxacin, norfloxacin, levofloxacin, tetracycline and TMP-SMZ	India	Spain	C
2011	Hassing [110]	Analysis of mechanisms involved in reduced susceptibility to ciprofloxacin in Salmonella enterica serotypes Typhi and Paratyphi A isolates from travellers to Southeast Asia	Salmonella enterica serotype Typhi and Salmonella enterica Paratyphi A	ciprofloxacin and nalidixic acid	India and Pakistan	Netherlands	A

2011	Hussenet [121]	Multidrug-resistant Acinetobacter baumannii infections in three returning travelers evacuated from Algeria, Thailand, and Turkey after hospitalization in local intensive care units	Acinetobacter baumannii	sulbactam, ticarcillin, ticarcillin/clavulanic acid, piperacillin, piperacillin/tazobactam, imipenem, meropenem, doripenem, ceftazidime, cefepime, cefpirome, tobramycin, isepamicin, gentamicin, netilmicin, chloramphenicol, tetracycline, pefloxacin, ofloxacin, ciprofloxacin, levofloxacin, rifampicin and TMP-SMZ	Algeria, Thailand and Turkey	France	A
2011	Le Hello [155]	International spread of an epidemic population of Salmonella enterica serotype Kentucky ST198 resistant to ciprofloxacin	Salmonella enterica serotype Kentucky	ciprofloxacin	Algeria, Cameroon, Djibouti, Egypt, Iran, Iraq, Jordan, Kenya, Lebanon, Libya, Mauritania, Morocco, Nigeria, Saudi Arabia, South Africa, Spain, Sudan, Syria, Tanzania, Togo, Tunisia and Turkey	France, UK and Denmark	C
2011	Medalla [166]	Ciprofloxacin-resistant Salmonella enterica Serotype Typhi, United States, 1999-2008	Salmonella enterica serotype Typhi	ampicillin, ciprofloxacin, chloramphenicol, TMP-SMZ, sulfamexazole, nalidixic acid, streptomycin and tetracycline	India and unspecified	USA	C
2011	Mina [172]	Canada's first case of a multi-resistant Corynebacterium diphtheriae strain, isolated from skin abscess	Corynebacterium diphtheriae	clindamycin, erythromycin, tetracycline, TMP-SMZ, ceftriaxone, cefotaxime, telithromycin and chloramphenicol	India	Canada	B

2011	O'Sullivan [190]	Burkholderia pseudomallei infection in a child with cystic fibrosis: Acquisition in the western hemisphere	Burkholderia pseudomallei	TMP-SMZ	Aruba	USA	C
2011	Ouyang-Latimer [191]	In vitro antimicrobial susceptibility of bacterial enteropathogens isolated from international travelers to Mexico, Guatemala, and India from 2006 to 2008.	Campylobacter spp. and Escherichia coli	ampicillin, nalidixic acid, tetracycline, doxycycline, TMP-SMZ, ceftriaxone, rifaximin, ciprofloxacin, levofloxacin and azithromycin	Guatemala, India and Mexico	USA	A
2011	Pandey [193] *	Travelers' diarrhea in Nepal: an update on the pathogens and antibiotic resistance	Shigella spp., Plesiomonas spp., Campylobacter spp., Aeromonas spp., Escherichia coli and Salmonella enterica spp. (Non-Typhoidal Serotypes)	amoxicillin, TMP-SMZ, nalidixic acid, ciprofloxacin and azithromycin	Nepal	North America, Australia, Europe, Japan and New Zealand	A
2011	Peirano [196]	New Delhi metallo-β-lactamase from traveler returning to Canada	Escherichia coli	amoxicillin/clavulanic acid, piperacillin/tazobactam, cefoxitin, ceftriaxone, ceftazidime, aztreonam, meropenem, ertapenem, amikacin, gentamicin, tobramycin, ciprofloxacin and TMP-SMZ	India	Canada	B
2011	Tappe [236]	Travel-related streptococcal toxic shock syndrome caused by emm type 78 Streptococcus pyogenes	Streptococcus pyogenes	tetracycline	Malaysia and Singapore	Germany	C

2012	Alexander [41]	Escherichia coli O104:H4 infections and international travel	Escherichia coli	amikacin, tetracycline, TMP-SMZ and extended-spectrum beta-lactams	Germany	Canada	A
2012	Ali [42]	Outbreak of a South West Pacific clone Panton-Valentine leucocidin-positive methicillin-resistant Staphylococcus aureus infection in a UK neonatal intensive care unit	Staphylococcus aureus	methicillin and erythromycin	Philippines	UK	B
2012	Flateau [94]	Recurrent pyelonephritis due to NDM-1 metallo-beta-lactamase producing Pseudomonas aeruginosa in a patient returning from Serbia, France, 2012	Pseudomonas aeruginosa	ticarcillin, ticarcillin/clavulanic acid, ceftazidime, cefepime, aztreonam, imipenem, meropenem, doripenem, tobramycin, gentamicin, amikacin, ciprofloxacin, doxycycline and tigecycline	Serbia	France	B
2012	Fleming [95]	Lactococcus garvieae multi-valve infective endocarditis in a traveler returning from South Korea	Lactococcus garvieae	ampicillin, gentamicin and clindamycin	South Korea	USA	B
2012	Hendriksen [113]	Characterization of isolates of Salmonella enterica serovar Stanley, a serovar endemic to Asia and associated with travel	Salmonella enterica serotype Stanley	amoxicillin, ampicillin, cefazolin, cefoxitin, cefpodoxime, ceftazidime, ceftiofur, ceftriaxone, cephalothin, chloramphenicol, ciprofloxacin, florfenicol, fosfomycin/trometamol, gentamicin, nalidixic acid, spectinomycin, streptomycin, sulfamethoxazole, tetracycline and trimethoprim	Afghanistan, Pakistan, Philippines, Thailand and Southeast Asia	Denmark and France	B

2012	Hrabak [116]	NDM-1 producing Acinetobacter baumannii isolated from a patient repatriated to the Czech Republic from Egypt, July 2011	Acinetobacter baumannii	ampicillin/sulbactam, amikacin, cefepime, ceftazidime, ciprofloxacin, gentamycin, meropenem, nalidixic acid, piperacillin, piperacillin/tazobactam, tetracycline and TMP-SMZ	Egypt	Czech Republic	B
2012	Jeon [129]	Quinolone-resistant Shigella flexneri isolated in a patient who travelled to India	Shigella flexneri	ampicillin/sulbactam, ampicillin, ciprofloxacin, norfloxacin, ofloxacin, levofloxacin and TMP-SMZ	India	South Korea	C
2012	Meyer [171]	Pet animals and foreign travel are risk factors for colonisation with extended-spectrum β-lactamase-producing Escherichia coli	Escherichia coli	beta-lactams	Greece and Africa	Germany	B
2012	Nemeth [181]	Multidrug-resistant bacteria in travellers hospitalized abroad: prevalence, characteristics, and influence on clinical outcome	Escherichia coli, Klebsiella pneumoniae, Acinetobacter baumannii, Enterobacter cloacae, Pseudomonas aeruginosa, Proteus spp., Staphylococcus aureus and other Enterobacteriaceae	amoxicillin/clavulanate, piperacillin/tazobactam, methicillin, carbapenems, cephalosporins, fluoroquinolones and aminoglycosides	Africa, Americas, Asia, Australia and Europe	Switzerland	B
2012	Niederer [182]	Genotypes and antibiotic resistances of Campylobacter jejuni and Campylobacter coli isolates from domestic and travel-associated human cases	Campylobacter jejuni and Campylobacter coli	quinolones	Europe and unspecified	Switzerland	C

2012	Rodriguez [209]	Potential international spread of multidrug-resistant invasive Salmonella enterica serovar Enteritidis	Salmonella enterica serotype Enteritidis	ampicillin, chloramphenicol, streptomycin, sulfonamides, tetracycline and trimethoprim	Nigeria and Uganda	UK	A
2012	Shiferaw [222]	Antimicrobial susceptibility patterns of Shigella isolates in foodborne diseases active Surveillance Network (FoodNet) Sites, 2000–2010	Shigella spp.	ampicillin, tetracycline, TMP-SMZ and chloramphenicol	Unspecified	USA	C
2012	Silva [226]	Outbreak of haemolytic uraemic syndrome due to Shiga toxin-producing Escherichia coli O104:H4 among French tourists returning from Turkey, September 2011	Escherichia coli	ampicillin, nalidixic acid, streptomycin, sulphonamide, trimethoprim, TMP-SMZ and tetracycline	Turkey	France	C
2012	Tatavarthy [237]	Molecular typing and resistance analysis of travel-associated Salmonella enterica serotype Typhi	Salmonella enterica serotype Typhi	amoxicillin/clavulanic acid, ampicillin, chloramphenicol, streptomycin, ciprofloxacin, nalidixic acid and TMP-SMZ	Bangladesh, Haiti, India, Pakistan, Peru and UAE	USA	C
2012	Tham [240]	Duration of colonization with extended-spectrum beta-lactamase-producing Escherichia coli in patients with travellers' diarrhoea	Escherichia coli	beta-Lactams	Afghanistan, Bangladesh, Bolivia, Cambodia, China, Cyprus, Egypt, Ghana, India, Iraq, Kurdistan, Pakistan, Spain, Syria, Lebanon, Thailand, Tunisia and UAE	Sweden	B

2013	Ahmed-Bentley [38]	Gram-negative bacteria that produce carbapenemases causing death attributed to recent foreign hospitalization	Klebsiella pneumoniae, Escherichia coli and Acinetobacter baumannii	amikacin, aztreonam, cefoxitin, ceftazidime, ceftriaxone, ciprofloxacin, ertapenem, gentamycin, imipenem, meropenem, piperacillin/tazobactam, tigecycline, TMP-SMZ and tobramycin	India	Canada	C
2013	Bathoorn [51]	Latent introduction to the Netherlands of multiple antibiotic resistance including NDM-1 after hospitalisation in Egypt, August 2013	Klebsiella pneumoniae	meropenem	Egypt	Netherlands	C
2013	Chan [68]	The characteristics of Klebsiella pneumoniae that produce KPC-2 imported from Greece	Klebsiella pneumoniae	amoxicillin/clavulanate, amikacin, aztreonam, cefoxitin, cefuroxime, ceftazidime, ciprofloxacin imipenem, meropenem, piperacillin/tazobactam, and tobramycin	Greece	Canada	C
2013	Decousser [79]	Outbreak of NDM-1-producing Acinetobacter baumannii in France, January to May 2013	Acinetobacter baumannii	ampicillin/sulbactam, ticarcillin/clavulanic acid, piperacillin, piperacillin/tazobactam, aztreonam, ceftazidime, cefepime, meropenem, imipenem, doripenem, ciprofloxacin, gentamycin, tobramycin, TMP-SMZ and fosfomycin	Algeria	France	C

2013	Hassing [109]	Decreased ciprofloxacin susceptibility in Salmonella Typhi and Paratyphi infections in ill-returned travellers: The impact on clinical outcome and future treatment options.	Salmonella enterica serotype Typhi and Salmonella enterica serotype Paratyphi A	ciprofloxacin	South Asia	Netherlands	B
2013	Josseaume [133]	Multidrug-resistant bacteria among patients treated in foreign hospitals: Management considerations during medical repatriation	Escherichia coli, Pseudomonas aeruginosa, Proteus mirabillis, Klebsiella pneumoniae, Enterococcus spp., Staphylococcus aureus, Acinetobacter baumannii, Enterobacteriaceae and unspecified	ceftazidime, methicillin vancomycin, beta-lactams, cephalosporins, quinolones and multidrug-resistant	Algeria, Congo, Morocco, Portugal, Spain, Thailand, Tunisia and Uruguay	France	C
2013	Ko [145]	First imported case of skin infection caused by PVL-positive ST30 community-associated methicillin-resistant Staphylococcus aureus clone in a returning Korean traveler from the Philippines	Staphylococcus aureus	methicillin	Philippines	South Korea	A
2013	Landelle [149]	Protracted outbreak of multidrug-resistant Acinetobacter baumannii after intercontinental transfer of colonized patients	Acinetobacter baumannii	ampicillin/sulbactam, ciprofloxacin, imipenem, rifampicin, aminoglycosides, cephalosporins and penicillins	Tahiti	France	B

2013	Lausch [154]	Colonisation with multi-resistant Enterobacteriaceae in hospitalised Danish patients with a history of recent travel: A cross-sectional study	Escherichia coli	beta-lactams	North Africa, Sub-Saharan Africa, South Asia, West Asia, Asia and Europe	Denmark	C
2013	Lee [156]	CTX-M-55-type extended-spectrum β-lactamase-producing Shigella sonnei isolated from a Korean patient who had travelled to China	Shigella sonnei	ampicillin, ampicillin/sulbactam, aztreonam, cefotaxime, ceftazidime, cephalothin, piperacillin and TMP-SMZ	China	South Korea	C
2013	Lo [159]	Complete sequence of an IncN plasmid, pIMP-HZ1, carrying blaIMP-4 in a Klebsiella pneumoniae strain associated with medical travel to China	Klebsiella pneumoniae	chloramphenicol, ertapenem, imipenem, meropenem, nitrofurantoin, TMP-SMZ and beta-lactams	China	Hong-Kong	C
2013	Mawatari [165]	Salmonella enterica serotype Paratyphi A carrying CTX-M-15 type extended-spectrum beta-lactamase isolated from a Japanese traveller returning from India, Japan, July 2013	Salmonella enterica serotype Paratyphi A	ampicillin, amoxicillin/clavulanic acid, aztreonam, cefotaxime, ceftazidime, ceftriaxone, ciprofloxacin, levofloxacin, nalidixic acid and TMP-SMZ	India	Japan	B
2013	Östholm-Balkhed [189]	Travel-associated faecal colonization with ESBL-producing Enterobacteriaceae: incidence and risk factors	Enterobacter cloacae, Escherichia coli, Klebsiella pneumoniae, and Proteus vulgaris	amikacin, amoxicillin/clavulanic acid, cefepime, cefotaxime, ceftazidime, ciprofloxacin, fosfomycin, gentamicin, mecillinam, nitrofurantoin, piperacillin/tazobactam, temocillin, tigecycline, TMP-SMZ, tobramycin and/or beta-lactams	North Africa, Sub-Saharan Africa, Central America, South America, South Asia and Asia	Sweden	B

2013	Paltansing [192]	Extended-spectrum β-lactamase–producing Enterobacteriaceae among travelers from the Netherlands	Enterobacteriaceae	beta-lactams	North Africa, Sub-Saharan Africa, Central America, South America, Southeast Asia, East Asia, South Asia, Central Asia and West Asia	Netherlands	C
2013	Pons [200]	Antimicrobial resistance in Shigella spp. causing traveller's diarrhoea (1995-2010): a retrospective analysis	Shigella spp.	ampicillin, amoxicillin/clavulanic acid, ciprofloxacin, Chloramphenicol, nalidixic acid, tetracycline and TMP-SMZ	North Africa, Sub-Saharan Africa, Central America, South America, Southeast Asia, East Asia, South Asia, Central Asia and West Asia	Spain	B
2013	Rogers [210]	Treatment options for new delhi metallo-beta-lactamase-harboring Enterobacteriaceae	Escherichia coli	amikacin, aztreonam, cefotaxime, cefoxitin, ceftazidime, ciprofloxacin, chloramphenicol, doripenem, fosfomycin, gentamicin, imipenem, meropenem, nitrofurantoin, piperacillin/tazobactam and TMP-SMZ	India	Australia	C
2013	Wang [254]	Carbapenem resistant Enterobacteriaceae carrying New Delhi metallo-β-lactamase gene (NDM-1) in Taiwan	Klebsiella pneumoniae and Klebsiella oxytoca	Amikacin, aztreonam, cefepime, ceftazidime, ciprofloxacin, doripenem, ertapenem, imipenem and meropenem	India and China	Taiwan	C

2014	Ageevets [36]	Emergence of carbapenemase-producing gram-negative bacteria in Saint Petersburg, Russia	Klebsiella pneumoniae	aztreonam, biapenem, cefepime, ciprofloxacin, cefepime/clavulanic acid, ertapenem, fosfomycin, gentamicin, imipenem, meropenem and polymyxin B	Vietnam	Russia	B
2014	Barlow [50]	Travel-associated antimicrobial drug–resistant nontyphoidal Salmonellae, 2004–2009	Salmonella enterica spp. (Non-Typhoidal Serotypes)	ampicillin, ceftriaxone, ciprofloxacin, gentamicin or TMP-SMZ	Canada, Mexico, Africa, Central America, South America, East Asia, Southeast Asia, Europe and Oceania	USA	B
2014	Birgand [54]	Introduction of highly resistant bacteria into a hospital via patients repatriated or recently hospitalized in a foreign country	Acinetobacter baumannii, Escherichia coli, Staphylococcus aureus, Enterococcus faecium, Enterobacter cloacae, Klebsiella pneumoniae and Proteus rettgeri	methicillin, bete-lactams, highly drug-resistant, Carbapenem-resistant and Glycopeptide-resistant	Algeria, Australia, Ecuador, Italy, Kuwait, Mali, Morocco, Portugal, Tunisia, USA and Vietnam	France	B
2014	Bodilsen [57]	Mycotic aneurysm caused by Burkholderia pseudomallei in a previously healthy returning traveller	Burkholderia pseudomallei	amoxicillin/clavulanate acid	Thailand	Denmark	C
2014	Cha [66]	Genetic diversity of Campylobacter jejuni isolates from Korea and travel-associated cases from east and southeast Asian countries	Campylobacter jejuni	ciprofloxacin, nalidixic acid and tetracycline	Indonesia, Philippines and China	South Korea	C

2014	Chua [71]	The growing burden of multidrug-resistant infections among returned Australian travellers	Klebsiella pneumoniae, Campylobacter spp., Comamonas spp., Pseudomonas aeruginosa and Escherichia coli	amikacin, amoxicillin/clavulanic acid, cefepime, ceftazidime, ceftriaxone, ciprofloxacin, gentamycin, meropenem, piperacillin/tazobactam, and TMP-SMZ	Afghanistan, Colombia, Croatia, Greece, India, Macedonia, Mauritius, Pakistan and Philippines	Australia	B
2014	Gunell [100]	Cefotaxime-resistant Salmonella enterica in travelers returning from Thailand to Finland	Salmonella enterica spp. (including serotypes Typhimurium, Senftenberg, Heidelberg, Concord, Minnesota and Grumpensis)	cefotaxime, beta-lactams and quinolones	China, Egypt, Ethiopia, Germany, India, Spain and Thailand	Finland	B
2014	Hashimoto [107]	Isolation of OXA-48 carbapenemase-producing Klebsiella pneumoniae ST101 from an overseas traveler returning to Japan	Klebsiella pneumoniae	amikacin, aztreonam, ceftazidime, cefazolin, cefmetazole, cefotaxime, gentamicin, imipenem/cilastatin, levofloxacin and piperacillin	Egypt and Turkey	Japan	B
2014	Hassing [108]	Salmonella subtypes with increased MICs for azithromycin in travelers returned to The Netherlands	Salmonella enterica serotype Typhi and Salmonella enterica Paratyphi	azithromycin and ciprofloxacin	Africa, East Asia, South Asia, Southeast Asia, West Asia and Europe	Netherlands	C
2014	Hopkins [27]	In vitro activity of rifaximin against clinical isolates of Escherichia coli and other enteropathogenic bacteria isolated from travellers returning to the UK	Escherichia coli, Shigella spp., Campylobacter spp., Salmonella enterica spp. (serotypes Typhi, Paratyphi and others)	azithromycin, ciprofloxacin, doxycycline, rifaximin, rifampicin and TMP-SMZ	North Africa, Sub-Saharan Africa, Central Asia, East Asia, South Asia, Southeast Asia, West Asia, Central America, South America, Europe and unspecified	UK	A

2014	Jorgensen [132]	High prevalence of faecal carriage of ESBL-producing Enterobacteriaceae in Norwegian patients with gastroenteritis	Enterobacteriaceae	beta-lactams	India, Pakistan, Africa, America, Asia and Europe	Norway	A
2014	Meltzer [167]	A large outbreak of Salmonella Paratyphi A infection among Israeli travelers to Nepal	Salmonella enterica serotype Paratyphi A	nalidixic acid	Nepal	Israel	C
2014	Nurjadi [20] +	Emergence of trimethoprim resistance gene dfrG in Staphylococcus aureus causing human infection and colonization in sub-Saharan Africa and its import to Europe	Staphylococcus aureus	Trimethoprim, Sulfamethoxazole and TMP-SMZ	Cameroon, Cape Verde, Democratic Republic of Congo, Gambia, Germany, Ghana, Guinea-Bissau, Kenya, Malawi, Mozambique, Nigeria, Rwanda, Senegal, Sierra Leone, South Africa, Sudan, Tanzania, Togo, Uganda and Zambia	France, Germany, Netherlands, Europe and unspecified	B
2014	O'Donnell [185]	Quinolone-resistant Salmonella enterica Serotype Enteritidis infections associated with international travel	Salmonella enterica serotype Enteritidis	nalidixic acid	China, Dominican Republic, France, Germany, Greece, India, Mexico, Philippines, Poland, Russia, Spain, UK and unspecified	USA	A
2014	Ricotta [207]	Epidemiology and antimicrobial resistance of international travel-associated Campylobacter infections in the United states, 2005-2011	Campylobacter spp.	quinolones and macrolides	China, Mexico, Sub-Saharan Africa, Central America, North America, South America, Asia and Europe	USA	B

2014	Sole [228]	Extended spectrum β-lactamase-producing Escherichia coli faecal carriage in Spanish travellers returning from tropical and subtropical countries	Escherichia coli	beta-lactams	Burkina Faso, Cameroon, Congo, Egypt, Equatorial Guinea, Ghana, Guatemala, India, Indonesia, Malaysia, Mali, Mexico, Morocco, Mozambique, Myanmar, Nepal, Nigeria, Peru, Senegal and Venezuela	Spain	B
2014	von Wintersdorff [252]	High rates of antimicrobial drug resistance gene acquisition after international travel, the Netherlands	Enterobacteriaceae	beta-lactams	Bangladesh, Bhutan, India, Nepal, Pakistan, Sri Lanka, North Africa, Sub-Saharan Africa, Central America, South America, South Asia, Southeast Asia and Europe	Netherlands	B
2014	Yaita [260]	Epidemiology of extended-spectrum β-lactamase producing Escherichia coli in the stools of returning Japanese travelers, and the risk factors for colonization	Escherichia coli	beta-lactams	India, Asia and Europe	Japan	B
2015	Allyn [43]	Delayed diagnosis of high drug-resistant microorganisms carriage in repatriated patients: three cases in a French intensive care unit	Enterobacter aerogenes, Acinetobacter baumannii, Klebsiella pneumoniae and Escherichia coli	carbapenems and beta-lactams	Madagascar	France	C

2015	Angue [46]	Risk factors for colonization with multidrug-resistant bacteria among patients admitted to the intensive care unit after returning from abroad	Escherichia coli, Klebsiella spp., Enterobacter spp., Acinetobacter baumanii, Pseudomonas aeruginosa, Enterobacteriaceae or Staphylococcus aureus	ceftazidime, methicillin, beta-lactams and/or carbapenems	Canada, China, Comoros, India, Madagascar, Mauritius, Southeast Asia or Europe	France	C
2015	Artzi [48]	Recurrent furunculosis in returning travelers: Newly defined entity	Staphylococcus aureus	methicillin	Argentina, Bolivia, Brazil, Chile, China, Costa Rica, Guatemala, Haiti, India, Laos, Mexico, Nicaragua, Panama, Peru, Sri Lanka, Thailand or Vietnam	Israel	B
2015	Baker [49]	Intercontinental dissemination of azithromycin-resistant shigellosis through sexual transmission: a cross-sectional study	Shigella flexneri	azithromycin	Algeria, Bangladesh, Burkina Faso, Cambodia, Democratic Republic of the Congo, Vietnam, Egypt, French Guiana, Ghana, India, Kenya, Maldives, Mali, Mexico, Morocco, Myanmar, Niger, Nigeria, Pakistan, Peru, South Africa, Tanzania, Togo, Uganda, Africa, Asia, Central America or South America	UK, France and Australia	C

2015	Bengtsson-Palme [52]	The Human Gut Microbiome as a Transporter of Antibiotic Resistance Genes between Continents	Escherichia coli	beta-lactams	India, Sri Lanka or Nepal	Sweden	C
2015	Bowen [60]	Importation and domestic transmission of Shigella sonnei resistant to ciprofloxacin - United States, May 2014-February 2015	Shigella sonnei	ampicillin, azithromycin, ciprofloxacin, nalidixic acid, streptomycin, sulfisoxazole, tetracycline and/or TMP-SMZ	Dominican Republic, Haiti, India, Morocco, Asia or Europe	USA	C
2015	Epelboin [85]	High rate of multidrug-resistant gram-negative bacillli carriage and infection in hospitalized returning travelers: A cross-sectional cohort study	Escherichia coli, Pseudomonas taiwanensis, Citrobacter braakii, Klebsiella oxytoca and Klebsiella pneumoniae	beta-lactams	Algeria, China, Côte d'Ivoire, Greece, Guadeloupe, Guinea, India, Malaysia, Mali, Nepal, Russia, Senegal, Spain, Tanzania, Thailand, Ukraine and USA	France	C
2015	FitzGerald [92]	Non-toxigenic penicillin-resistant cutaneous C. diphtheriae infection: A case report and review of the literature	Corynebacterium diphtheriae	penicillin	Ethiopia	UK	C
2015	Hendriksen [114]	Genomic dissection of travel-associated extended-spectrum-beta-lactamase-producing Salmonella enterica serovar typhi isolates originating from the Philippines: A one-off occurrence or a threat to effective treatment of typhoid fever?	Salmonella enterica serotype Typhi	ampicillin, cefotaxime, cefpodoxime, ceftazidime, ceftiofur, ceftriaxone, gentamicin, streptomycin, tetracycline and trimethoprim	Philippines	Netherlands	C

2015	Huber [119]	Primary skin melioidosis in a returning traveler	Burkholderia pseudomallei	TMP-SMZ	Thailand	Germany	A
2015	Kantele [134]	Antimicrobials increase travelers risk of colonisation by extended-spectrum betalactamase-producing Enterobacteriaceae	Enterobacteriaceae	beta-lactams	North Africa, Sub-Saharan Africa, East Asia, South Asia, Southeast Asia and West Asia	Finland	B
2015	Kaspar [137]	Colonization with resistant microorganisms in patients transferred from abroad: Who needs to be screened?	Staphylococcus aureus or Gram-negative bacteria	ceftazidime, cefotaxime, methicillin, beta-lactams and/or carbapenems	France, Italy, Spain, Switzerland, Africa, Americas, Asia, Europe and unspecified	Switzerland	A
2015	Kim [140]	Outbreak of ciprofloxacin-resistant Shigella sonnei associated with travel to Vietnam, Republic of Korea	Shigella sonnei	ampicillin, ampicillin/sulbactam, amoxicillin/clavulanate, cefotaxime, cefoxitin, ceftazidime, ceftriaxone, cephalothin, ciprofloxacin, nalidixic acid, streptomycin, tetracycline and TMP-SMZ.	Vietnam	South Korea	C
2015	Lane [150]	Travel destinations and sexual behavior as indicators of antibiotic resistant Shigella strains - Victoria, Australia	Shigella sonnei, Shigella flexneri, Shigella boydii, and Shigella dysenteriae	ampicillin, ciprofloxacin, nalidixic acid, spectinomycin, streptomycin, sulphathiozole, tetracycline and trimethoprim	India, Indonesia, Thailand, North Africa, Sub-Sahara Africa, Americas, Central Asia, Southern Asia, Southeast Asia, West Asia, Oceania and unspecified	Australia	A

2015	Lubbert [160] +	Colonisation with extended-spectrum beta-lactamase-producing and carbapenemase-producing Enterobacteriaceae in international travelers returning to Germany	Escherichia coli and Klebsiella pneumoniae	amikacin, ampicillin/sulbactam, aztreonam, ceftazidime, ceftibuten, ciprofloxacin, colistin, doripenem, doxycycline, ertapenem, fosfomycin, gentamicin, levofloxacin, imipenem, meropenem, moxifloxacin, piperacillin/tazobactam, tigecycline, tobramycin, and/or TMP-SMZ	Argentina, Benin, Bolivia, Botswana, Brazil, Cambodia, Cameroon, Chile, China, Colombia, Democratic Republic of the Congo, Costa Rica, Côte d'Ivoire, Cuba, Ecuador, Ethiopia, Fiji, France, Ghana, Guatemala, India, Indonesia, Italy, Kenya, Laos, Malaysia, Mexico, Moldova, Mozambique, Myanmar, Namibia, Nepal, New Zealand, Nicaragua, Panama, Paraguay, Peru, Philippines, Portugal, Seychelles, Singapore, South Africa, Sri Lanka, Swaziland, Tanzania, Thailand, Togo, Turkey, Uganda, USA, Venezuela, Vietnam or Zimbabwe	Germany	B

2015	Mutters [178]	Influx of multidrug-resistant organisms by country-to-country transfer of patients	Acinetobacter baumanii, Enterobacter cloacae, Enterococcus faecium, Escherichia coli, Klebsiella pneumoniae, Klebsiella oxytoca, Proteus mirabilis, Pseudomonas aeruginosa and Staphylococcus aureus	methicillin, vancomycin, beta-lactams and/or carbapenams	Russia, Sub-Saharan Africa, North Africa, West Asia, Asia or Europe	Germany	C
2015	Nuesch-Inderbinen [183]	Antimicrobial susceptibility of travel-related Salmonella enterica serovar Typhi isolates detected in Switzerland (2002-2013) and molecular characterization of quinolone resistant isolates	Salmonella enterica serotype Typhi	ampicillin, amoxicillin/clavulanic acid, cephalothin, chloramphenicol, ciprofloxacin, nalidixic acid, streptomycin, sulfamethoxazole, tetracycline and trimethoprim	Australia, Bangladesh, Cambodia, Costa Rica, India, Lebanon, Mexico, Nepal, Niger, Pakistan, Sri Lanka, Tanzania, Africa and Asia	Switzerland	C

2015	Nurjadi [21]	Skin and soft tissue infection in intercontinental travellers and the import of multi-resistant Staphylococcus aureus to Europe	Staphylococcus aureus	ciprofloxacin, clindamycin, erythromycin, methicillin, tetracycline and TMP-SMZ	Australia, Bahamas, Benin, Brazil, Burundi, Cambodia, Cameroon, Cape Verde, Columbia, Democratic Republic of Congo, Republic of Congo, Costa Rica, Cuba, Dominican Republic, Ecuador, Equatorial Guinea, Fiji, Gambia, Ghana, Guinea , issau, Haiti, India, Indonesia, Jamaica, Kenya, Laos, Madagascar, Malawi, Malaysia, Mexico, Morocco, Mozambique, Namibia, Nepal, New Zealand, Nicaragua, Niger, Nigeria, Pakistan, Panama, Papua New Guinea, Paraguay, Peru, Philippines, Rwanda, Senegal, Sierra Leone, Singapore, South Africa, Sri Lanka, Sudan, Surinam, Tanzania, Thailand, Togo, Tunisia, Uganda, Vietnam or Zambia	Europe	C

2015	Nurjadi [22]	Predominance of dfrG as determinant of trimethoprim resistance in imported Staphylococcus aureus	Staphylococcus aureus	trimethoprim and TMP-SMZ	Germany and the same as the above, Nurjadi et. al., 2015.	Europe	B
2015	Ruppe [212] +	High rate of acquisition but short duration of carriage of multidrug-resistant Enterobacteriaceae after travel to the tropics	Enterobacteriaceae	beta-lactams	Angola, Argentina, Bangladesh, Benin, Bolivia, Brazil, Burkina Faso, Cambodia, Cameroon, China, Congo, Costa Rica, Ecuador, Ethiopia, French Guyana, Gabon, Ghana, Guinea, India, Indonesia, Ivory Coast, Kenya, Laos, Madagascar, Malaysia, Mali, Mexico, Myanmar, Nepal, Niger, Peru, Senegal, Sri Lanka, Tanzania, Thailand, Togo, Venezuela and Vietnam	France, Sub-Saharan Africa, Central America, South America and Asia	B
2015	Salazar-Austin [215]	Extensively drug-resistant tuberculosis in a young child after travel to India	Mycobacterium tuberculosis	amikacin, ethambutol, isoniazid, kanamycin, moxifloxacin, ofloxacin, pyrazinamide, rifabutin, rifampicin and streptomycin	India	USA	C

2015	Shin [223]	First report and molecular characterization of a Campylobacter jejuni isolate with extensive drug resistance from a travel-associated human case	Campylobacter jejuni	amikacin, ampicillin, azithromycin, cefotaxime, ceftazidime, chloramphenicol, ciprofloxacin, clindamycin, enrofloxacin, erythromycin, gentamicin, nalidixic acid, streptomycin and tetracycline	Philippines	South Korea	A
2015	Tojo [241]	Multidrug-resistant Acinetobactor baumannii isolated from a traveler returned from Brunei	Acinetobacter baumannii	amikacin, arbekacin, aztreonam, cefepime, cefotaxime, cefoxitin, ceftazidime, cephradine, ciprofloxacin, colistin, fosfomycin, gentamicin, imipenem, meropenem, ofloxacin, piperacillin and piperacillin/tazobactam	Brunei	Japan	C
2015	Trojanek [242]	Enteric fever imported to the Czech Republic: epidemiology, clinical characteristics and antimicrobial susceptibility	Salmonella enterica serotype Typhi and Salmonella enterica serotype Paratyphi A	ampicillin, ciprofloxacin, chloramphenicol and TMP-SMZ	Angola, India and Nepal	Czech Republic	C
2015	Valverde [245]	CTX-M-15-non-ST131 Escherichia coli isolates are mainly responsible of faecal carriage with ESBL-producing Enterobacteriaceae in travellers, immigrants and those visiting friends and relatives	Escherichia coli	amikacin, ciprofloxacin, gentamycin, nalidixic acid, sulfonamide, tobramycin, TMP-SMZ, trimethoprim and beta-lactams	Angola, Bolivia, Cambodia, Colombia, Cuba, Dominican Republic, Ecuador, Ethiopia, Guatemala, India, Indonesia, Kenya, Mali, Nepal, Peru, Philippines, Sudan, Thailand and Venezuela	Spain	B
2016	Ahmad Hatib [37]	Enteric fever in a tertiary paediatric hospital: A retrospective six-year review	Salmonella enterica serotype Typhi	ampicillin, ciprofloxacin and/or TMP-SMZ	Bangladesh, India, Indonesia, Malaysia, Nepal or Pakistan	Singapore	C

2016	Bernasconi [53] +	Travelers can import colistin-resistant Enterobacteriaceae, including those possessing the plasmid-mediated mcr-1 gene	Escherichia coli, Klebsiella pneumoniae and Proteus mirabilis	colistin, polymyxin B and cephalosporins	India and Switzerland	India and Switzerland	B
2016	Date [76]	Changing patterns in enteric fever incidence and increasing antibiotic resistance of enteric fever isolates in the United States, 2008-2012	Salmonella enterica serotype Typhi and Salmonella enterica serotype Paratyphi A	ampicillin, ceftriaxone, chloramphenicol, ciprofloxacin, nalidixic acid and TMP-SMZ	Bangladesh, Brazil, Burundi, Cambodia, China, El Salvador, Ghana, Guatemala, Guinea, Haiti, Iceland, India, Iraq, Lebanon, Liberia, Mali, Mexico, Nepal, Nigeria, Pakistan, Philippines, Seri Lanka, Tanzania, Togo, Zimbabwe and Central America	USA	C
2016	Frickmann [96]	Low enteric colonization with multidrug-resistant pathogens in Soldiers returning from deployments - Experience from the years 2007–2015	Escherichia coli and Enterococcus faecalis	ciprofloxacin, gentamycin, levofloxacin, nitrofurantoin, streptomycin, teicoplanin, TMP-SMZ, vancomycin, beta-lactams and cephalosporins	Afghanistan, Democratic Republic of Congo, Djibouti, Ghana, Lebanon, Mali, Nigeria, South Sudan, Sub-Saharan Africa, Sudan, Tanzania, Thailand, Uganda, Uzbekistan and unspecified	Germany	C
2016	Herrera-Leon [115]	Plasmid-mediated quinolone resistance in different diarrheagenic Escherichia coli pathotypes responsible for complicated, noncomplicated, and traveler's diarrhea cases	Escherichia coli	ampicillin, amoxicillin/clavulanic acid, cefotaxime, cephalothin, chloramphenicol, ciprofloxacin, tetracycline, streptomycin, TMP-SMZ and sulfonamides	Mexico and Southeast Asia	Spain	C

2016	Jamal [128]	High prevalence of New Delhi metallo-β-lactamase-1 (NDM-1) producers among carbapenem-resistant Enterobacteriaceae in Kuwait	Enterobacter cloacae, Escherichia coli, Klebsiella pneumoniae and Morganella morganii	amikacin, amoxicillin/clavulanic acid, cefepime, cefotaxime, ceftriaxone, ciprofloxacin, clarithromycin, colistin, imipenem, meropenem, metronidazole, nitrofurantoin, piperacillin/tazobactam, tigecycline and vancomycin	Egypt, Jordan, Syria, USA, Iran and India	Kuwait	B
2016	Kong [147]	Melioidosis acquired by a traveler from Papua New Guinea	Burkholderia pseudomallei	ampicillin, ciprofloxacin, gentamicin, tobramycin and piperacillin/tazobactam	Papua New Guinea	China	C
2016	Li [157]	Surveillance for travel and domestically acquired multidrug-resistant human Shigella infections - Pennsylvania, 2006-2014	Shigella flexneri, Shigella sonnei, Shigella boydii, and Shigella dysenteriae	ampicillin, amoxicillin/clavulanic acid, cefoxitin, ceftiofur, ceftriaxone, chloramphenicol, ciprofloxacin, gentamicin, nalidixic acid, streptomycin, sulfisoxazole, tetracycline and TMP-SMZ	Aruba, Belize, Burundi, Canada, China, Colombia, Costa Rica, Dominican Republic, Ecuador, Egypt, El Salvador, France, Ghana, Guatemala, Haiti, Hawaii, Honduras, India, Iraq, Israel, Jamaica, Mexico, Morocco, Nepal, Nicaragua, Niger, Nigeria, Pakistan, Panama, Peru and Thailand	USA	B

2016	Mataseje [164]	Colistin-nonsusceptible Pseudomonas aeruginosa sequence Type 654 with blaNDM-1 arrives in North America	Escherichia coli, Providencia rettgeri and Pseudomonas aeruginosa	amikacin, cefepime, cefoxitin, ceftazidime, ceftriaxone, ciprofloxacin, colistin, ertapenem, gentamicin, meropenem, piperacillin/tazobactam, tigecycline and tobramycin	India	Canada	A
2016	Reuland [206]	Prevalence and risk factors for carriage of ESBL-producing Enterobacteriaceae in Amsterdam	Enterobacter cloacae, Escherichia coli, Klebsiella pneumoniae or Serratia plymuthica	beta-lactams, ciprofloxacin, gentamycin, nitrofurantoin and TMP-SMZ	Afghanistan, Canada, China, Egypt, India, Iran, Israel, Japan, Mongolia, Morocco, Sri Lanka, Turkey, UAE, USA, Sub-Saharan Africa, Central America, South America and Southeast Asia	Netherlands	C
2016	Saitoh [214]	Increase in paratyphoid fever cases in Japanese travellers returning from Cambodia in 2013	Salmonella enterica serotype Paratyphi A	nalidixic acid	Cambodia	Japan	B
2016	Sekirov [219]	Epidemiologic and genotypic review of carbapenemase-producing organisms in British Columbia, Canada, between 2008 and 2014	Enterobacter spp., Escherichia coli and Klebsiella pneumoniae	Carbapenem	Bangladesh, India and unspecified	Canada	B
2016	Senok [220]	Diversity of methicillin-resistant Staphylococcus aureus CC22-MRSA-IV from Saudi Arabia and the Gulf region	Staphylococcus aureus	ampicillin, imipenem, methicillin and penicillin	Egypt	Saudi Arabia	A

2016	Strysko [230]	International travel is a risk factor for extended-spectrum β-lactamase-producing Enterobacteriaceae acquisition in children: A case-case-control study in an urban U.S. hospital	Enterobacteriaceae	beta-lactams, ciprofloxacin, nitrofurantoin and/or TMP-SMZ	Bangladesh, Canada, China, Pakistan, Peru and Saudi Arabia	USA	B
2016	Talan [233]	Fluoroquinolone-resistant and extended-spectrum β-lactamase-producing Escherichia coli infections in patients with pyelonephritis, United States	Escherichia coli	ampicillin, cefazolin, ceftriaxone, ciprofloxacin, levofloxacin, gentamicin, TMP-SMZ and/or beta-lactams	Mexico, Central America and Asia	USA	B
2016	Von Dach [251]	Comparative genomics of community-associated methicillin-resistant Staphylococcus aureus shows the emergence of clone ST8-USA300 in Geneva, Switzerland	Staphylococcus aureus	ciprofloxacin, clindamycin, erythromycin, flucloxacillin/oxacillin, methicillin, penicillin G and tetracycline	Bolivia, Brazil, Cameroon, Columbia, Cuba, Ecuador, Sierra Leone, Switzerland, Uruguay and USA	Switzerland	C
2017	Adelman [34]	Ovarian endometrioma superinfected with Salmonella: Case report and review of the literature	Salmonella enterica serotype Schwarzengrund	ciprofloxacin and levofloxacin	UAE	USA	C
2017	Ageevets [35]	Genetic environment of the blaKPC-2 gene in a Klebsiella pneumoniae isolate that may have been imported to Russia from Southeast Asia	Klebsiella pneumoniae	ampicillin, aztreonam, biapenem, cefepime, cefotaxime, ceftazidime, ciprofloxacin, chloramphenicol, ertapenem, fosfomycin, gentamicin, imipenem, meropenem, polymyxin B and TMP-SMZ	Vietnam	Russia	A

2017	Arcilla [18]	Import and spread of extended-spectrum β-lactamase-producing Enterobacteriaceae by international travellers (COMBAT study): a prospective, multicentre cohort study	Enterobacteriaceae	beta-lactams	Brazil, China, Egypt, Gambia, Ghana, India, Indonesia, Kenya, Malaysia, Mexico, Morocco, Myanmar, Nepal, Peru, South Africa, Sri Lanka, Suriname, Tanzania, Thailand, Turkey, Uganda, Vietnam, North Africa, Sub-Saharan Africa, Central America, North America, South America, Central Asia, East Asia, Southeast Asia, South Asia, West Asia, Europe and Oceania	Netherlands	B
2017	Blomfeldt [55]	Emerging multidrug-resistant Bengal Bay clone ST772-MRSA-V in Norway: molecular epidemiology 2004-2014	Staphylococcus aureus	cefoxitin, clindamycin, erythromycin, fusidic acid, gentamicin, methicillin, mupirocin, norfloxacin, rifampicin, tetracycline and/or TMP-SMZ	Africa, Asia, Europe and unspecified	Norway	B
2017	Cusumano [73]	Rapidly growing Mycobacterium infections after cosmetic surgery in medical tourists: the Bronx experience and a review of the literature	Mycobacterium abscessus and Mycobacterium chelonae/abscessus complex	cefoxitin, imipenem/cilastatin, and linezolid	Dominican Republic	USA	B

2017	Dave [77]	What were the risk factors and trends in antimicrobial resistance for enteric fever in London 2005-2012?	Salmonella enterica serotype Typhi and Salmonella enterica serotype Paratyphi A	ampicillin, ciprofloxacin, chloramphenicol, nalidixic acid, sulphonamide, trimethoprim and TMP-SMZ	Bangladesh, India, Pakistan, Africa and Asia	UK	B
2017	Day [78]	Antimicrobial resistance in Shiga toxin-producing Escherichia coli serogroups O157 and O26 isolated from human cases of diarrhoeal disease in England, 2015	Escherichia coli	chloramphenicol, aminoglycosides, beta-lactams, fluoroquinolones, macrolides, sulphonamides, tetracyclines and trimethoprim	Bangladesh, Belgium, India, Morocco, Portugal, Spain, Turkey, Europe and unspecified	UK	B
2017	Di Ruscio [81]	MRSA infections in Norway: A study of the temporal evolution, 2006-2015	Staphylococcus aureus	methicillin	Brazil, Cuba, Egypt, Eritrea, Ethiopia, Greece, India, Pakistan, Philippines, Poland, Somalia, Sri Lanka, Spain, Thailand, Turkey, UK, Africa, Central America, North America, South America, Asia, Europe, Oceania and unspecified	Norway	C
2017	Ferstl [90]	Severe infection with multidrug-resistant Salmonella Choleraesuis in a young patient with primary sclerosing cholangitis	Salmonella enterica serotype Choleraesuis	beta-lactams and fluoroquinolones	Thailand	Germany	B
2017	Geissler [97]	Increasing Campylobacter infections, outbreaks, and antimicrobial resistance in the United States, 2004-2012	Campylobacter spp.	ciprofloxacin and erythromycin	unspecified	USA	C

2017	Inkster [124]	First outbreak of colonization by linezolid- and glycopeptide-resistant Enterococcus faecium harbouring the cfr gene in a UK nephrology unit	Enterococcus faecium, Escherichia coli and Pseudomonas aeruginosa	linezolid and carbapenem	India	UK	B
2017	Kantele [135]	Fluoroquinolone antibiotic users select fluoroquinolone-resistant ESBL-producing Enterobacteriaceae (ESBL-PE) - Data of prospective traveller study	Enterobacteriaceae	amoxicillin/clavulanic acid, ampicillin, cefalexin, cefepime, ceftatzidime, ceftriaxone, cefuroxime, ciprofloxacin, colistin, ertapenem, levofloxacin, nitrofurantoin, piperacillin/tazobactam, TMP-SMZ and tobramycin	North Africa, Sub-Saharan Africa, East Asia, South Asia, Southeast Asia and West Asia	Finland	B
2017	Khawaja [139]	Patients hospitalized abroad as importers of multiresistant bacteria - a cross-sectional study	Staphylococcus aureus, Acinetobacter baumannii, Pseudomonas aeruginosa, Enterococci spp. and Enterobacteriaceae	ceftatzidime, meropenem, methicillin, vancomycin, beta-lactams, carbapenems	North Africa, Sub-Saharan Africa, East Asia, Southeast Asia, South Asia, West Asia, Central America, North America, South America, Europe and Oceania	Finland	B
2017	Mischlinger [173]	Dalbavancin for outpatient parenteral antimicrobial therapy of skin and soft tissue infections in a returning traveller: Proposal for novel treatment indications	Staphylococcus aureus	methicillin	China, Philippines and Singapore	Austria	B
2017	Muchena [175]	Determinants of multidrug resistance among previously treated tuberculosis patients in Zimbabwe, 2014	Mycobacterium tuberculosis	Isoniazid and rifampicin	Botswana, South Africa and Zambia	Zimbabwe	A

2017	Ny [184]	Community carriage of ESBL-producing Escherichia coli is associated with strains of low pathogenicity: A Swedish nationwide study.	Escherichia coli	amoxicillin/clavulanic acid, ampicillin, cefotaxime, ceftazidime, ciprofloxacin, colistin, fosfomycin, gentamicin, imipenem, mecillinam, piperacillin/tazobactam, tobramycin and beta-lactams	Denmark, Finland, Iceland, Norway, Sweden, Africa, North America, South America, Asia and/or Europe	Sweden	C
2017	Post [203]	Antibiotic susceptibility profiles among Campylobacter isolates obtained from international travelers between 2007 and 2014	Campylobacter spp.	azithromycin, ciprofloxacin, erythromycin, levofloxacin and tetracycline	Burkina Faso, Cameroon, China, Cuba, DRC, Ethiopia, France, India, Indonesia, Morocco, Nepal, Nicaragua, Pakistan, Peru, Poland, Romania, South Africa, Spain, Tanzania, Tunis, Turkey, USA, North Africa, Sub-Saharan Africa, Central America, North America, South America, East Asia, South Asia, Southeast Asia, West Asia and Europe	Belgium	A
2017	Principe [204]	First report of NDM-1-producing Klebsiella pneumoniae imported from Africa to Italy: Evidence of the need for continuous surveillance	Acinetobacter baumannii and Klebsiella pneumoniae	amikacin, amoxicillin/clavulanic acid, cefepime, cefotaxime, ceftazidime, ciprofloxacin, ertapenem, gentamicin, imipenem, meropenem, piperacillin/tazobactam, TMP-SMZ and carbapenem	Egypt	Italy	C

2017	Reinheimer [205]	Prevalence of multidrug-resistant organisms in refugee patients, medical tourists and domestic patients admitted to a German university hospital	Acinetobacter baumannii, Escherichia coli, Klebsiella pneumoniae, Pseudomonas aeruginosa, Staphylococcus aureus and Enterobacteriaceae	methicillin, piperacillin, beta-lactams, carbapenems, cephalosporins and fluoroquinolones	Afghanistan, Algeria, Croatia, Egypt, Eritrea, Ethiopia, Ghana, Greek, Hungary, India, Iran, Iraq, Italy, Kuwait, Morocco, Nigeria, Saudi Arabia, Somalia, Spain, Sri Lanka, Thailand, Turkey and unspecified	Germany	C
2017	Rieber [208]	Molecular investigation of carbapenem-resistant Acinetobacter spp. from hospitals in North Rhine-Westphalia, Germany	Acinetobacter baumannii	ciprofloxacin, gentamicin and carbapenms	Libya	Germany	C

2017	Sadouki [213]	Comparison of phenotypic and WGS-derived antimicrobial resistance profiles of Shigella sonnei isolated from cases of diarrhoeal disease in England and Wales, 2015	Shigella sonnei	ampicillin, azithromycin, cefotaxime, ceftazidime, chloramphenicol, ciprofloxacin, gentamicin, streptomycin, sulphonamide, tetracycline and trimethoprim	Afghanistan, Bangladesh, Barbados, Bermuda, Cape verde, China, Colombia, Cuba, Dominican Republic, Egypt, France, Georgia, Ghana, Guatemala, India, Indonesia, Kenya, Lebanon, Malawi, Mali, Mexico, Morocco, Nepal, Pakistan, Peru, Philippines, Saudi Arabia, Somalia, Sudan, Tanzania, Thailand, Turkey, USA, Uzbekistan, Vietnam, Sub-Saharan Africa, Central America, North America, South America and unspecified	UK	C
2017	Samuelsen [216]	Molecular and epidemiological characterization of carbapenemase-producing Enterobacteriaceae in Norway, 2007 to 2014	Citrobacter spp., Enterobacter cloacae complex, Escherichia coli, Klebsiella pneumoniae, Klebsiella quasipneumoniae, Klebsiella variicola, Proteus mirabilis and Providencia stuartii	amikacin, aztreonam, cefotaxime, ceftazidime, cefuroxime, ciprofloxacin, colistin, ertapenem, fosfomycin, gentamicin, imipenem, mecillinam, meropenem, piperacillin/tazobactam, temocillin, tigecycline, TMP-SMZ, tobramycin and carbapenem	Brazil, Greece, India, Jamaica, Jordan, Morocco, Pakistan, Romania, Serbia, Spain, Sri Lanka, Syria, Thailand, Turkey, UAE, USA and unspecified	Norway	B

2017	Wielders [256]	Extended-spectrum β-lactamase- and pAmpC-producing Enterobacteriaceae among the general population in a livestock-dense area	Escherichia coli and Klebsiella pneumoniae	beta-lactams	Australia, New Zealand, Turkey, North Africa, Sub-Saharan Africa, North America, Central America, South America, Central Asia, East Asia, South Asia, Southeast Asia, West Asia and Europe	Netherlands	B
2018	Allyn [44]	Medical evacuation from abroad of critically ill patients	Citrobacter freundii, Enterococcus faecium and Klebsiella pneumoniae	imipenem, meropenem, vancomycin, beta-lactams and carbapenems	Mauritius	Reunion Island	C
2018	Brown [62]	CTX-M-65 extended-spectrum β-lactamase–producing Salmonella enterica serotype Infantis, United States	Salmonella enterica serotype Infantis	ampicillin, cefoxitin, ceftiofur, ceftriaxone, chloramphenicol, ciprofloxacin, gentamicin, nalidixic acid, streptomycin, sulfisoxazole, tetracycline and TMP-SMZ	Ecuador and Peru	USA	B
2018	Espenhain [86]	Travel to Asia is a strong predictor for carriage of cephalosporin resistant E. coli and Klebsiella spp. but does not explain everything; Prevalence study at a Norwegian hospital 2014-2016	Escherichia coli and Klebsiella pneumoniae	cefotaxime, ceftazidime, piperacillin-tazobactam, gentamicin, cephalosporins and/or fluoroquinolones	Thailand, Turkey, Pakistan, Africa, America, Asia and Europe	Norway	B

2018	Espinal [87]	First description of blaNDM-7 carried on an IncX4 plasmid in Escherichia coli ST679 isolated in Spain	Escherichia coli and Staphylococcus aureus	amikacin, amoxicillin/clavulanic acid, ampicillin, aztreonam, cefepime, cefotaxime, cefoxitin, ceftazidime, ciprofloxacin, doripenem, ertapenem, gentamicin, imipenem, levofloxacin, meropenem, methicillin, nalidixic acid, piperacillin/tazobactam, tetracycline, ticarcillin, tobramycin, TMP-SMZ and carbapenems	Pakistan	Spain	B
2018	Flateau [93]	Prevalence and risk factors for extended-spectrum beta-lactamase-producing-Enterobacteriaceae in French military and civilian travelers: A cross-sectional analysis	Escherichia coli and/or Klebsiella pneumoniae	beta-lactams	Afghanistan, Australia, Cambodia, Cameroon, Canary Islands, Central African Republic, France, French Guyana, India, Israel, Ivory Coast, Lebanon, Mali, Mauritius, Saudi Arabia, Thailand, UAE, Vietnam, Africa, Central America, North America, South America, Asia and Europe	France	B
2018	Hebbelstrup [112]	Characterization of diarrheagenic enteroaggregative Escherichia coli in Danish adults - Antibiotic treatment does not reduce duration of diarrhea	Escherichia coli	ampicillin, azithromycin, cefotaxime, ceftazidime, , chloramphenicol, ciprofloxacin, gentamycin, nalidixic acid, sulfamethoxazole, tetracycline and/or trimethoprim	Cuba, Egypt, Germany, India, Nepal, Tanzania, Thailand and/or Turkey	Denmark	A

2018	Islam [125]	Intestinal carriage of third-generation cephalosporin-resistant and extended-spectrum β-lactamase-producing Enterobacteriaceae in healthy US children	Escherichia coli or Klebsiella pneumoniae	cefepime, ciprofloxacin, gentamicin, meropenem, piperacillin/tazobactam, TMP-SMZ, beta-lactams and/or cephalosporines	Mexico, Nepal, Dominican Republic and Vietnam	USA	C
2018	Johansen [131]	Whole-genome sequencing and antimicrobial resistance in Brucella melitensis from a Norwegian perspective	Brucella melitensis	Rifampicin	Afghanistan, Ethiopia, Georgia, Iraq, Israel, Somalia and Turkey	Norway	C
2018	Karp [136]	Plasmid-mediated quinolone resistance in human non-typhoidal Salmonella infections: An emerging public health problem in the United States	Salmonella enterica spp. (Non-Typhoidal Serotypes)	ampicillin, azithromycin, ceftiofur, ceftriaxone, chloramphenicol, ciprofloxacin, gentamicin, kanamycin, streptomycin, sulfisoxazole, tetracycline and TMP-SMZ	Cambodia, China, Dominican Republic, Egypt, Ethiopia, Indonesia, Israel, Malaysia, Mexico, Philippines, Taiwan, Thailand and Vietnam	USA	C
2018	Klemm [143]	Emergence of an extensively drug-resistant Salmonella enterica serovar Typhi Clone harboring a promiscuous plasmid encoding resistance to fluoroquinolones and third-generation cephalosporins	Salmonella enterica serotype Typhi	ceftriaxone	Pakistan	UK	C

2018	Knaapila [144]	Antibiotic susceptibility of intestinal Escherichia coli in men undergoing transrectal prostate biopsies: a prospective, registered, multicentre study	Escherichia coli	fluoroquinolone	Denmark, Estonia, Finland, Germany, Iceland, Japan, Latvia, Lithuania, Poland, Russia, Sweden, North Africa, Sub-Saharan Africa, North America, South America, Asia and/or Europe	Finland	C
2018	Kohler [146]	Emergence of carbapenemase-producing Enterobacteriaceae, South-Central Ontario, Canada	Citrobacter spp., Enterobacter spp., Escherichia coli, Klebsiella pneumoniae, Morganella morganii, Serratia marcescens, Klebsiella oxytoca, Providencia rettgeri and/or Proteus mirabilis	amikacin, ciprofloxacin, colistin, ertapenem, fosfomycin, gentamicin, imipenem, meropenem, nitrofurantoin, tigecyclin, tobramycin, TMP-SMZ and/or carbapenems	Austria, Bangladesh, Barbados, Bhutan, Brazil, China, Croatia, Dominican Republic, Egypt, France, Germany, Greece, India, Israel, Italy, Jamaica, Maldives, Nepal, Pakistan, Philippines, Portugal, Saudi Arabia, Spain, Sri Lanka, Thailand, Turkey, USA and/or Vietnam	Canada	B
2018	Macaux [161]	Extensively-drug-resistant bacteria carriers among overseas travellers: one-third had not been hospitalized previously	Acinetobacter baumannii, Citrobacter amaloniticus, Citrobacter freundii, Enterobacter cloacae, Enterococcus faecium, Escherichia coli and/or Klebsiella pneumoniae	vancomycin or carbapenems	India, Morocco, USA, Sub-Saharan Africa, Southeast Asia, West Asia and Europe	France	C

2018	Mataseje [163]	Characterization of OXA-48-like carbapenemase producers in Canada, 2011-14	Escherichia coli or Klebsiella pneumoniae	amikacin, amoxicillin/clavulanic acid, ampicillin, cefazolin, cefotaxime, cefoxitin, cefpodoxime, ceftazidime, eftriaxone, ciprofloxacin, colistin, ertapenem, gentamicin, meropenem, nitrofurantoin, piperacillin/tazobactam, tigecycline, TMP-SMZ, tobramycin and/or beta-lactams	Egypt, Lebanon, Libya, Nigeria, Pakistan, Saudi Arabia, Syria, UAE, Ukraine, USA and South Asia	Canada	B
2018	Mittal [174]	Two for the price of one: Emerging carbapenemases in a returning traveller to New York City	Klebsiella pneumoniae	amikacin, ampicillin, ampicillin-sulbactam, aztreonam, cefazolin, cefoxitin, ceftriaxone, cefepime, ceftazidime-avibactam, chloramphenicol, ciprofloxacin, gentamicin, meropenem, meropenem-vaborbactam, piperacillin/tazobactam, polymyxin B, tigecycline, tobramycin and TMP-SMZ	Bangladesh	USA	C
2018	Mulvey [176]	Characterization of a colistin-resistant Salmonella enterica 4,[5],12: I: - Harbouring mcr-3.2 on a variant IncHI-2 plasmid identified in Canada	Salmonella enterica serotype 4,[5],12:i:-	aztreonam, cefotaxime, ceftazidime, cefepime, chloramphenicol, ciprofloxacin, colistin, gentamicin, kanamycin, polymyxin B, tetracycline and TMP-SMZ	Thailand	Canada	B
2018	Pommelet [199]	Enteric fever among children: 50 cases in a French tertiary care centre	Salmonella enterica serotype Typhi and Salmonella enterica serotype Paratyphi	ampicillin, ciprofloxacin chloramphenicol, nalidixic acid and TMP-SMZ	Bangladesh, Cameroon, India and Pakistan	France	C

2018	Terry [239]	Antimicrobial resistance profiles of Shigella dysenteriae isolated from travellers returning to the UK, 2004-2017	Shigella dysenteriae	ciprofloxacin, chloramphenicol, streptomycin, tetracycline, trimethoprim, beta-lactams and sulphonamides	Afghanistan, Bangladesh, Cape verde, Congo, Egypt, Ethiopia,Gambia, Ghana, India, Iraq, Kenya, Malawi, Morocco, Nepal, Nigeria, Pakistan, Panama, Somalia, South Africa, Sudan, Tanzania, Turkey, United Arab Emirates, Zimbabwe, Sub-Saharan Africa and unspecified	UK	B
2018	Ukah [243]	Risk factors for acquisition of multidrug-resistant Escherichia coli and development of community-acquired urinary tract infections	Escherichia coli	amoxicillin/clavulanic acid, ampicillin, azithromycin, cefoxitin, ceftiofur, ceftriaxone, chloramphenicol, ciprofloxacin, gentamicin, kanamycin, nalidixic acid, streptomycin, sulfisoxazole, tetracycline and/or TMP-SMZ	Asia and unspecified	Canada	B
2018	Valentin [244]	Proteus mirabilis harboring carbapenemase NDM-5 and ESBL VEB-6 detected in Austria	Escherichia coli, Klebsiella pneumoniae and Proteus mirabilis	ceftazidim/avibactam, ceftolozan/tazobactam, colistin, fosfomycin, meropenem, piperacillin/tazobactam and tigecycline	Bangladesh	Austria	B

2018	Walker [253]	A cluster of multidrug-resistant Mycobacterium tuberculosis among patients arriving in Europe from the Horn of Africa: A molecular epidemiological study	Mycobacterium tuberculosis	capreomycin, ethambutol, isoniazid, pyrazinamide and rifampicin	Djibouti, Eritrea, Ethiopia and Somalia and Sudan	Austria, Finland, France, Germany, Sweden, Switzerland and UK	B
2018	Williams [257]	A case of multi-drug resistant ESBL-producing Shigella sonnei acute acalculous cholecystitis and gastroenteritis in a returned traveller	Shigella sonnei	amoxicillin, azithromycin, ceftriaxone, TMP-SMZ and beta-lactams	Malaysia and Vietnam	Australia	B
2018	Williamson [258]	Increasing antimicrobial resistance in nontyphoidal Salmonella Isolates in Australia from 1979 to 2015	Salmonella enterica spp. (Non-Typhoidal Serotypes)	ampicillin, cefotaxime, chloramphenicol, ciprofloxacin, gentamicin, kanamycin, nalidixic acid, streptomycin, sulphathiozole, tetracycline and/or trimethoprim	Indonesia, Malaysia, Thailand, Vietnam and unspecified	Australia	C
2019	Boyd [61]	Results from the Canadian nosocomial infection surveillance program for detection of carbapenemase-producing Acinetobacter spp. in Canadian hospitals, 2010-16	Acinetobacter baumannii, Acinetobacter bereziniae, Acinetobacter pittii and/or Acinetobacter soli	amikacin, ceftazidime, ceftriaxone, ciprofloxacin, colistin, gentamicin, meropenem, piperacillin/tazobactam, tigecycline, tobramycin, TMP-SMZ	China, South Korea, North Africa, Sub-Saharan Africa, South Asia, Central America, Europe and unspecified	Canada	C
2019	Chatham-Stephens [69]	Emergence of extensively drug-resistant Salmonella Typhi infections among travelers to or from Pakistan - United States, 2016-2018	Salmonella enterica serotype Typhi	ampicillin, chloramphenicol, TMP-SMZ, cephalosporins and fluoroquinolones	Pakistan	USA	A

2019	Dall [74]	Do probiotics prevent colonization with multi-resistant Enterobacteriaceae during travel? A randomized controlled trial	Enterobacteriaceae	carbapenems and/or beta-lactams	Denmark and India	Denmark and India	B
2019	Engsbro [84]	Ceftriaxone-resistant Salmonella enterica serotype Typhi in a pregnant traveller returning from Karachi, Pakistan to Denmark, 2019	Escherichia coli and Salmonella enterica serotype Typhi	ampicillin, aztreonam, cefpodoxime, ceftriaxone, ciprofloxacin, TMP-SMZ and carbapenems	Pakistan	Denmark	B
2019	Eyre [89]	Detection in the United Kingdom of the Neisseria gonorrhoeae FC428 clone, with ceftriaxone resistance and intermediate resistance to azithromycin, October to December 2018	Neisseria gonorrhoeae	azithromycin, cefixime, ceftriaxone, ciprofloxacin, penicillin and tetracycline	Spain	UK	C
2019	Haley [104]	Salmonella enterica serovar Kentucky recovered from human clinical cases in Maryland, USA (2011–2015)	Salmonella enterica serovar Kentucky	aminoglycosides, beta-lactams and sulfonamides	North Africa, South Asia and Europe	USA	C
2019	Hanrahan [105]	Emergence and spread of ciprofloxacin-resistant Neisseria gonorrhoeae in New South Wales, Australia: lessons from history	Neisseria gonorrhoeae	ciprofloxacin	Asia and unspecified	Australia	B

2019	Huang [118]	Methicillin-resistant Staphylococcus aureus nasal carriage in international medical conference attendees	Staphylococcus aureus	methicillin	Australia, Bangladesh, Canada, China, Denmark, France, Hong Kong, India, Indonesia, Iraq, Japan, Korea, Malaysia, Mauritius, Philippines, Saudi Arabia, Singapore, Sri Lanka, Swiss, Thailand, UK, USA and Vietnam	Taiwan	A
2019	Huynh [122]	Multidrug-resistant tuberculous meningitis in a returned traveller	Mycobacterium tuberculosis	rifampicin	Pakistan	Australia	C
2019	Ingle [123]	Co-circulation of multidrug-resistant Shigella among men who have sex with men, Australia	Shigella flexneri and Shigella sonnei	ampicillin, azithromycin, ceftriaxone, ciprofloxacin, gentamicin, sulfonamide and trimethoprim	North Africa, Sub-Saharan Africa, North Asia, South Asia, Southeast Asia, West Asia, Americas, Europe, Oceania and unspecified	Australia	C
2019	Klein [142]	Increase in the prevalence of Panton-Valentine leukocidin and clonal shift in community-onset methicillin-resistant Staphylococcus aureus causing skin and soft-tissue infections in the Rhine-Neckar Region, Germany, 2012-2016	Staphylococcus aureus	clindamycin, fusidic acid, methicillin, mupirocin, tetracycline, TMP-SMZ, fluoroquinolones and macrolides	unspecified	Germany	C
2019	Langelier [151]	Microbiome and Antimicrobial Resistance Gene Dynamics in International Travelers	Escherichia coli	beta-lactam	Nepal, Nigeria, Uganda and USA	Nepal and USA	B

2019	Nurjadi [23]	Import of community-associated, methicillin-resistant Staphylococcus aureus to Europe through skin and soft-tissue infection in intercontinental travellers, 2011-2016	Staphylococcus aureus	methicillin	Australia, North Africa, Sub-Saharan Africa, South Asia, Southeast Asia, West Asia, Central America, South America and Oceania	Europe	C
2019	Pires [197]	Gut microbiota dynamics in travelers returning from India colonized with extended-spectrum cephalosporin-resistant Enterobacteriaceae: A longitudinal study	Enterobacteriaceae	beta-lactams	India and Switzerland	India and Switzerland	B
2019	Schaumburg [217] +	Acquisition and colonization dynamics of antimicrobial-resistant bacteria during international travel: a prospective cohort study	Enteric Bacteria (includes Aeromonas caviae, Aeromonas hydrophila, Aeromonas sobria, Aeromonas veronii, Comamonas testosteroni, Enterobacter cloacae complex, Escherichia coli, Raoultella ornithinolytica)	colistin, beta-lactams and carbapenems	Bolivia, Brazil, Cambodia, China, Costa Rica, Dominican Republic, Egypt, Ecuador, Germany, India, Indonesia, Myanmar, Nepal, Netherlands, Panama, Peru, Rwanda, Singapore, Thailand, Vietnam, Africa, Asia, North America, South America and Europe	Germany, Netherlands and unspecified	C
2019	Siira [225]	Whole genome sequencing of Salmonella Chester reveals geographically distinct clusters, Norway, 2000 to 2016	Salmonella enterica serovar Chester	chloramphenicol, tetracycline, TMP-SMZ, beta-lactams and quinolones	Cyprus, Greece, Ivory Coast, Morocco, Senegal, Sri Lanka and Thailand	Norway	B

2019	Skjøt-Arkil [227]	Carrier prevalence and risk factors for colonisation of multiresistant bacteria in Danish emergency departments: a cross-sectional survey	Enterobacter cloacae, Escherichia coli, Citrobacter spp., Klebsiella spp. and/or Staphylococcus aureus	methicillin, vancomycin, beta-lactams and/or carbapenams	Africa, North America, South America, Asia, Europe and Oceania	Denmark	C

#, Grade A was given to randomised control trials or for scoring 75% or above (in reference to the maximum score) on a Newcastle-Ottawa Scale based or adapted assessment protocol, grade B for 50-74%, and C was for less than 50%; +, studies containing antimicrobial resistant isolates pre- or before travel; *, studies containing antimicrobial resistant organisms isolated during travelling; TMP-SMZ, trimethoprim/sulfamethoxazole; MDR, multidrug resistance: including studies that mentioned multidrug resistant organisms; USA, United States of America; UK, United Kingdom; UAE, United Arab Emirates.

Tropical Medicine and Infectious Disease

Table S2. List of Countries or regions that are documented in the review.

Countries/regions mentioned in literature	Categorized as
Angola	Sub-Saharan Africa
Benin	
Botswana	
Burkina Faso	
Burundi	
Cameroon	
Cape Verde	
Central Africa	
Central African Republic	
Comoros	
Congo	
Democratic Republic of the Congo	
Equatorial Guinea	
Eritrea	
Ethiopia	
French Guiana	
Gabon	
Gambia	
Ghana	
Guinea	
Guinea-Bissau	
Ivory Coast / Côte d'Ivoire	
Kenya	
Liberia	
Madagascar	
Malawi	
Mali	
Mauritius	
Mozambique	
Niger	
Nigeria	
Republic of the Congo	
Reunion Island	
Rwanda	
Senegal	
Seychelles	
Sierra Leone	
Somalia	
South Sudan	
Tanzania	
Togo	
Uganda	
Zambia	
Zimbabwe	
Africa	
East Africa / Eastern Africa	

Middle Africa<br>Sahara<br>South Africa / Southern Africa<br>Sub-Saharan Africa<br>West Africa	
Algeria<br>Cyprus<br>Egypt<br>Iran<br>Iraq<br>Israel<br>Jordan<br>Kuwait<br>Lebanon<br>Maghreb / Morocco<br>Saudi Arabia<br>Sudan<br>Syria<br>Tunisia<br>Turkey<br>UAE / United Arab Emirates<br>Arab countries<br>Dead Sea<br>Middle East<br>North Africa<br>Western Asia	West Asia and North Africa
Kurdistan<br>Uzbekistan<br>Central Asia	Central Asia
China<br>Hong Kong<br>Japan<br>Mongolia<br>North Korea<br>South Korea<br>Taiwan<br>East Asia<br>Far East Asia	East Asia
Russia	North Asia
Afghanistan<br>Bangladesh<br>Bhutan<br>India<br>Maldives<br>Myanmar<br>Nepal<br>Pakistan<br>Sri Lanka<br>Indian peninsula/Indian subcontinent<br>South Asia/Southern Asia	South Asia

Southern Central Asia	
Brunei<br>Cambodia<br>Indonesia<br>Laos<br>Malaysia<br>Philippines<br>Singapore<br>Thailand<br>Vietnam<br>Southeast Asia	Southeast Asia
Aruba<br>Bahamas<br>Barbados<br>Belize<br>Bolivia<br>Brazil<br>Caribbean<br>Colombia<br>Costa Rica<br>Cuba<br>Dominican Republic<br>Ecuador<br>El Salvador<br>Guadeloupe<br>Guatemala<br>Haiti<br>Jamaica<br>Mexico<br>Nicaragua<br>Panama<br>Paraguay<br>Peru<br>Suriname<br>Uruguay<br>Venezuela<br>Central America<br>Latin America<br>South America<br>Tropical South America	Central and South America
Canada<br>USA<br>Bermuda<br>Northern America	North America
Austria<br>Belgium<br>Croatia<br>Czech Republic<br>Denmark<br>Estonia	Europe

Europe	
Finland	
France	
Georgia	
Germany	
Great Britain / England / UK	
Greece	
Holland / Netherlands	
Hungary	
Iceland	
Ireland	
Italy	
Macedonia	
Moldova	
Norway	
Poland	
Portugal	
Romania	
Serbia	
Spain	
Sweden	
Switzerland	
Ukraine	
Yugoslavia	
Nordic	
North-eastern Mediterranean	
Australia	Oceania
Hawaii	
New Guinea	
New Zealand	
Papua New Guinea	
Tahiti	
Antarctica	
Oceania	
Pacific (islands)	
(Multiple categories or not mentioned/specified)	Other

Table S3. Top originating regions for AMR organisms (source).

#	Region	Isolates	Studies	##	Top Locations	Isolates	Studies
1	South Asia	6551	116	1	India	3602	70
				2	Pakistan	501	31
				3	Nepal	580	15
2	Southeast Asia	2243	77	1	Thailand	1012	34
				2	Philippines	173	18
				3	Vietnam	215	17
3	East Asia	564	34	1	China	109	21
				2	Japan	4	3
				3	Koreas	3	2
4	North Africa and West Asia	1368	81	1	Egypt	292	28
				2	Turkey	191	19
				3	Morocco	181	16
5	Sub-Saharan Africa	3082	96	1	Kenya	1176	12
				2	Tanzania	44	11
				3	Nigeria	15	8
6	Central and South America	3205	59	1	Jamaica	363	4
				2	Mexico	620	21
				3	Brazil	47	9
7	Europe	2047	70	1	Spain	386	21
				2	Greece	96	13
				3	UK	38	5
8	North America	398	25	1	USA	128	16
				2	Canada	10	5
9	Oceania	58	13	1	Australia	5	2
				2	Tahiti	2	1
10	Other *	8520	63	1	Unspecified	6138	48
				2	Multiple	2382	18

*, Travelling from multiple regions, or no mention of travel source; AMR, Antimicrobial resistant.

Table S4. Top destination regions for AMR organisms (destination).

#	Region	Isolates	Studies	##	Top Locations	Isolates	Studies
1	Europe	15229	138	1	UK	3656	22
				2	Spain	1993	17
				3	Finland	3382	10
2	North America	9261	57	1	USA	5449	40
				2	Canada	3812	17
3	Oceania	1650	11	1	Australia	1650	11
4	East Asia	461	18	1	Japan	423	9
				2	South Korea	22	6
				3	Taiwan	14	2
5	Other	2962	4	1	Unspecified*	2472	3

*, Travelling from multiple regions, or no mention of travel destination; AMR, Antimicrobial resistant.

Table S5. Number of studies and isolates for species that were documented in the analyzed studies.

#	Species	Number of documenting travelling AMR	
		studies	isolates
01	Acinetobacter spp.*	21	187
02	Aeromonas spp.*	3	108
03	Brucella melitensis	1	14
04	Burkholderia pseudomallei	5	5
05	Campylobacter spp.*	19	3281
06	Citrobacter spp.*	3	3
07	Comamonas spp.	1	1
08	Corynebacterium diphtheriae	2	2
09	Enterobacter spp.*	9	49
10	Enterococcus spp.*	8	40
11	Escherichia coli*	59	5461
12	Klebsiella spp.*	28	207
13	Lactococcus garvieae	1	1
14	Morganella morganii*	1	1
15	Mycobacterium spp.	5	91
16	Neisseria gonorrhoeae	3	120
17	Plesiomonas spp.*	2	39
18	Proteus spp.*	7	14
19	Providencia spp.*	2	2
20	Pseudomonas spp. *	12	44
21	Raoultella ornithinolytica*	1	1
22	Salmonella spp.*	63	6032
23	Shigella spp.*	29	6931
24	Staphylococcus aureus	35	2162
25	Streptococcus pyogenes	1	1
26	Vibrio spp.*	3	5
27	enteric bacteria (not specified) *	29	5200
28	Other organisms (not specified)	3	58
	Total		30060

*, enteric bacteria.

Table S6. Numbers of travel-related AMR isolates documented in 238 studies, up to June 2019 inclusive, categorized by source and isolation time.

All organisms	As					NAf&WAs					SSAf					Eu				
Antimicrobial resistance component (Number of isolates)	Before 1990	1990-1999	2000-2009	2010-2019	Total	Before 1990	1990-1999	2000-2009	2010-2019	Total	Before 1990	1990-1999	2000-2009	2010-2019	Total	Before 1990	1990-1999	2000-2009	2010-2019	Total
Any AMR (30060)	16	2150	5943	1965	10799 a.b	0	179	790	399	1368	0	1600	849	633	3082	0	451	1023	573	2047
Beta-lactams: All (10474)	16	605	2187	1558	4366	0	17	254	294	565	0	348	428	404	1180	0	171	543	471	1185
Beta-lactams: Penicillins (6320)	16	564	1623	278	2481	0	16	172	50	238	0	347	364	52	763	0	169	503	57	729
Beta-lactams: Carbapenems (1922)	0	249	266	276	791	0	0	16	64	80	0	186	174	108	468	0	100	71	133	304
Beta-lactams: Cephalosporins (2100)	0	291	420	231	942	0	1	32	34	67	0	187	249	40	476	0	102	81	51	234
Macrolides and Licosamides (848)	0	53	133	139	325	0	4	1	2	7	0	5	4	30	39	0	1	5	15	21
Quinolones (9213)	0	907	3241	447	4710 [1]	0	32	399	116	547	0	250	338	56	644	0	214	525	58	797
Sulfonamides and Trimethoprim (7268)	16	721	1294	466	2499	0	42	100	136	279	0	668	368	172	1208	0	236	84	40	360
Tetracyclines (5095)	0	683	551	206	1440	0	36	79	66	181	0	597	269	84	951	0	207	75	16	298
Aminoglycosides (4200)	0	533	549	561	1363	0	14	52	113	179	0	412	250	107	769	0	102	92	30	224
Amphenicol (2633)	16	458	503	97	1074	0	4	4	21	29	0	248	227	35	510	0	102	50	5	157

All organisms	NAm					S&CAm					Oc					Other				
Antimicrobial resistance component (Number of isolates)	Before 1990	1990-1999	2000-2009	2010-2019	Total	Before 1990	1990-1999	2000-2009	2010-2019	Total	Before 1990	1990-1999	2000-2009	2010-2019	Total	Before 1990	1990-1999	2000-2009	2010-2019	Total
Any AMR (30060)	25	0	326	47	398	354	716	2343	363	3776	0	0	41	17	58	679	1462	5373	1006	8520
Beta-lactams: All (10474)	5	0	157	41	203	30	186	488	265	969	0	0	30	7	37	157	212	1061	539	1969
Beta-lactams: Penicillins (6320)	5	0	138	11	154	30	186	450	63	729	0	0	30	7	37	157	210	631	191	1189
Beta-lactams: Carbapenems (1922)	0	0	12	3	15	0	48	55	67	170	0	0	2	0	2	0	0	13	79	92
Beta-lactams: Cephalosporins (2100)	0	0	9	5	14	0	48	87	31	166	0	0	3	0	3	0	2	94	102	196
Macrolides and Licosamides (848)	0	0	1	6	7	0	7	88	18	113	0	0	1	4	5	15	60	134	122	331
Quinolones (9213)	0	0	45	36	81	0	95	563	101	760	0	0	3	2	5	18	33	1236	382	1669
Sulfonamides and Trimethoprim (7268)	8	0	104	31	143	113	251	588	97	1049	0	0	10	8	18	235	282	1034	10	1712
Tetracyclines (5095)	6	0	38	5	49	78	232	571	46	927	0	0	1	1	2	110	344	678	115	1247
Aminoglycosides (4200)	5	0	78	31	114	84	129	259	58	530	0	0	3	2	5	174	219	493	130	1016
Amphenicol (2633)	1	0	9	0	10	51	97	126	19	293	0	0	0	0	0	66	192	232	70	560

Others, predefined or unspecified (1861)	0	97	130	137	364	0	0	25	88	113	0	27	18	105	150	0	0	38	28	66	0	0	33	30	63	0	31	84	19	134	0	0	3	1	4	0	10	910	47	967
MDR (4290)	16	539	693	371	2229 [2]	0	15	29	95	139	0	276	218	108	602	0	102	73	63	238	0	0	1	5	6	57	127	72	73	330	0	0	3	5	8	37	203	286	212	738

[1], includes 115 isolates with a broad isolation time frame, 1984–2015; [2], includes 610 isolates with a broad isolation time frame, 1984–2015; MDR, multidrug resistant organisms; organisms documented as multidrug resistant or resistant to three or more classes of antimicrobials; SSAf, Sub-Saharan Africa, includes isolates originated from Africa but not specified from which country or area; NAf&Was, West Asia and North Africa; As, Asia, includes isolates originated from Asia but not specified from which country or area, and does not include isolates documented specifically from West Asia; Eu: Europe; Nam, North America; S&Cam, Central and South America, includes isolates originated from Americas but not specified from which country or area; Oc, Oceania; Other, unspecified or multiple regions were documented.

24

Table S7. Number of travel-related isolates for enteric organisms with documented AMR component.

Antimicrobial resistance component (Number of isolates)		As					NAf&WAs					SSAf					Eu					NAm					S&CAm					Oc					Other				
		Before 1990	1990-1999	2000-2009	2010-2019	Total	Before 1990	1990-1999	2000-2009	2010-2019	Total	Before 1990	1990-1999	2000-2009	2010-2019	Total	Before 1990	1990-1999	2000-2009	2010-2019	Total	Before 1990	1990-1999	2000-2009	2010-2019	Total	Before 1990	1990-1999	2000-2009	2010-2019	Total	Before 1990	1990-1999	2000-2009	2010-2019	Total	Before 1990	1990-1999	2000-2009	2010-2019	Total
Any AMR (27593)		16	2042	5206	1734	9725a&B	0	179	647	369	1195	0	1600	789	426	2816	0	451	591	520	1562	25	0	236	42	303	354	716	2253	298	3623	0	0	14	10	24	679	1439	5331	896	8345
Beta-lactams	All (8587)	16	605	1450	1475	3546	0	17	113	278	408	0	348	370	394	1112	0	171	112	442	725	5	0	67	36	108	30	186	399	232	847	0	0	3	5	8	157	212	1019	445	1833
	Penicillins (5336)	16	566	1073	383	2038	0	16	54	79	150	0	347	327	121	795	0	169	103	146	418	5	0	63	6	74	30	186	387	93	696	0	0	3	5	8	157	212	742	46	1157
	Carbapenems (1454)	0	249	266	101	616	0	0	16	29	45	0	186	174	12	372	0	100	71	46	217	0	0	12	2	14	0	48	55	17	120	0	0-	2	0	2	0	0	13	55	68
	Cephalosporins (1848)	0	290	390	153	833	0	1	32	34	67	0	187	248	10	445	0	102	68	44	214	0	0	9	5	14	0	48	86	23	157	0	0	3	0	3	0	0	93	22	115
Macrolides and Licosamides (493)		0	53	103	27	183	0	4	1	2	7	0	5	3	2	10	0	1	5	7	13	0	0	0	2	2	0	7	87	4	98	0	0	0	1	1	15	59	75	30	179
Quinolones (8779)		0	799	3211	330	4457 [1]	0	32	399	108	539	0	250	337	21	608	0	214	525	47	786	0	0	45	32	77	0	95	563	90	749	0	0	3	0	3	18	22	1234	286	1560
Sulfonamides and Trimethoprim (6848)		16	721	1264	321	2324	0	42	100	128	271	0	668	367	57	1092	0	236	84	22	342	8	0	104	30	142	133	251	587	82	1033	0	0	10	8	18	235	282	1033	76	1626
Tetracyclines (4799)		0	683	520	110	1313	0	36	79	58	173	0	597	268	35	900	0	207	74	10	291	6	0	38	3	47	38	232	571	37	878	0	0	1	0	1	110	335	677	34	1156
Aminoglycosides (3892)		0	553	519	163	1235	0	14	52	103	169	0	412	249	48	710	0	102	92	23	217	5	0	78	30	113	84	129	258	52	523	0	0	4	0	4	174	207	492	48	921
Amphenicol (2489)		16	458	503	50	1027	0	4	4	21	29	0	248	227	11	486	0	102	50	3	155	1	0	9	0	10	51	97	126	17	291	0	0	0	0	0	66	192	232	1	491

Others, predefined or unspecified (3097)	0	97	99	308	1114 [2]	0	0	19	91	110	0	27	15	138	180	0	0	27	139	166	0	0	31	28	59	0	31	84	76	191	0	0	2	1	3	0	130	967	177	1274
MDR (4083)	16	539	662	313	2140 [2]	0	15	29	86	130	0	276	217	73	566	0	102	72	29	203	0	0	0	5	5	57	127	71	68	323	0	0	2	4	6	37	194	285	194	710

[1], includes 115 isolates with a broad isolation time frame, 1984–2015; [2], includes 610 isolates with a broad isolation time frame, 1984–2015; MDR, multidrug resistant organisms; organisms documented as multidrug resistant or resistant to three or more classes of antimicrobials; SSAf, Sub-Saharan Africa, includes isolates originated from Africa but not specified from which country or area; NAf&Was, West Asia and North Africa; As, Asia, includes isolates originated from Asia but not specified from which country or area, and does not include isolates documented specifically from West Asia; Eu: Europe; Nam, North America; S&Cam, Central and South America, includes isolates originated from Americas but not specified from which country or area; Oc, Oceania; Other, unspecified or multiple regions were documented.

Table S8. Antimicrobials categories that were included in the analysis.

Antimicrobial class		Mentioned antimicrobial Name	
beta-lactams	penicillins	ampicillin/sulbactam, amoxicillin/clavulanate, flucloxacillin/oxacillin, isoxapenicillin, mecillinam, oxacillin, penicillin G, piperacillin/tazobactam, piperacillin, temocillin, ticarcillin, ticarcillin/clavulanic acid, and studies mentioning penicillins with no specification	and studies mentioning beta-lactams with no specification
	carbapenems	biapenem, doripenem, ertapenem, imipenem, imipenem/cilastatin, meropenem-vaborbactam, meropenem, and studies mentioning carbapenems with no specification	
	cephalosporins	cefepime, cefotaxime, cefoxitin, ceftazidime, ceftiofur, ceftriaxone, cefazolin, cefmetazole, cefalexin, cefuroxime axetil, cefaclor, cefepime/clavulanic acid, cefradine, cefpodoxime, cephalothin, and studies mentioning cephalosporins with no specification	
macrolides and lincosamides		azithromycin, clarithromycin, clindamycin, erythromycin, telithromycin, and studies mentioning macrolides and lincosamides with no specification	
quinolones		ciprofloxacin, denalidixic acid, enrofloxacin, levofloxacin, nalidixic acid, Norfloxacin, ofloxacin, pefloxacin, and studies mentioning quinolones with no specification	
sulfonamides and trimethoprim		trimethoprim, TMP-SMZ, sulfamethoxazole, sulfisoxazole, sulphathiozole, and studies mentioning sulfonamides with no specification	
tetracyclines		doxycycline, tetracycline, tigecycline	
aminoglycosides		amikacin, arbekacin, capreomycin, gentamicin, isepamicin, kanamycin, netilmicin, neomycin, streptomycin, spectinomycin, tobramycin, and studies mentioning aminoglycosides with no specification	
amphenicol		chloramphenicol, florfenicol	
Others/not classified		aztreonam, colistin, ethambutol, fosfomycin, fosfomycin/trometamol, fucidic acid, furazolidone, isoniazid, linezolid, metronidazole, mupirocin, nitrofurantoin, oxazolidinones, polymyxin B, pyrazinamide, rifabutin, rifampicin, teicoplanin, vancomycin	

© 2019 by the authors. Submitted for possible open access publication under the terms and conditions of the Creative Commons Attribution (CC BY) license (http://creativecommons.org/licenses/by/4.0/).
